# CYP1B1 modulates stress and repair pathways in airway cells challenged by wood smoke particles

**DOI:** 10.1093/toxsci/kfag003

**Published:** 2026-01-25

**Authors:** Lili Sun, Marysol Almestica-Roberts, Nam D Nguyen, Jacob Cowley, Erin Gail Romero, Samantha N Serna, Peng Zhang, Changshan Niu, Tosifa A Memon, Cassandra E Deering-Rice, Christopher A Reilly

**Affiliations:** Department of Pharmacology and Toxicology, Center for Human Toxicology, University of Utah, Salt Lake City, UT 84112, United States; Department of Pharmacology and Toxicology, University of Utah, Salt Lake City, UT 84112, United States; Department of Pharmacology and Toxicology, University of Utah, Salt Lake City, UT 84112, United States; Department of Pharmacology and Toxicology, University of Utah, Salt Lake City, UT 84112, United States; Department of Pharmacology and Toxicology, Center for Human Toxicology, University of Utah, Salt Lake City, UT 84112, United States; Department of Pharmacology and Toxicology, University of Utah, Salt Lake City, UT 84112, United States; Department of Pharmacology and Toxicology, University of Utah, Salt Lake City, UT 84112, United States; Department of Pharmacology and Toxicology, University of Utah, Salt Lake City, UT 84112, United States; Department of Pharmacology and Toxicology, University of Utah, Salt Lake City, UT 84112, United States; Department of Pharmacology and Toxicology, Center for Human Toxicology, University of Utah, Salt Lake City, UT 84112, United States; Department of Pharmacology and Toxicology, Center for Human Toxicology, University of Utah, Salt Lake City, UT 84112, United States

**Keywords:** CYP1B1, airway epithelial cells, wood smoke particulate matter, ERS, NRF2

## Abstract

Environmental pollution negatively impacts respiratory health by damaging and reprogramming airway epithelial cells (AECs). CYP1B1 is one of the most highly induced genes in AECs exposed to combustion-derived air pollutants such as wood smoke particulate matter (WSPM) and plays dual roles in generating toxic reactive intermediates and in the detoxification of xenobiotics of diverse nature. However, the significance of CYP1B1 induction by AECs challenged with pollutants remains unclear. A comparison of BEAS-2B and CYP1B1-overexpressing BEAS-2B cells revealed that CYP1B1 overexpression reduced acute cytotoxicity and enhanced proliferation and migration following WSPM-induced injury in vitro. Conversely, inhibition of CYP1B1 in HBEC3-KT cells increased cytotoxicity and decreased proliferation. CYP1B1 inhibition in HBEC3-KT cells exacerbated endoplasmic reticulum stress (ERS), which promotes cell cycle arrest and cytotoxicity, while overexpression of CYP1B1 attenuated ERS. CYP1B1 Inhibition also enhanced the expression of mRNA for the NRF2 target genes NQO1 and HMOX1, and the proinflammatory cytokine IL8, whereas CYP1B1 overexpression downregulated mRNA expression for NQO1 and HMOX1. In vivo, *Cyp1b1*-deficient mice exhibited greater basal lung inflammation, but limited response to WSPM-treatment compared with wild-type mice. However, *Cyp1b1^−/−^* derived mouse tracheal epithelial cells treated with WSPM showed a more pronounced inflammatory response, characterized by exacerbated *Cxcl1*, *Cxcl2*, and *Trpa1* mRNA expression compared with wild-type cells. In conclusion, CYP1B1 mitigates WSPM-induced damage to AECs by squelching ERS, oxidative stress, NRF2, and inflammatory signaling, thereby supporting cellular defense and repair. Additional interactions with CYP1A1 and TRP channels also suggest a broader role in AEC physiology.

Air pollution threatens human health on a global scale. Wood smoke particulate matter (WSPM) is a specific and pervasive air pollutant composed of solids and condensed chemicals with variable pneumotoxic potential (Nguyen et al. 2020; Burrell et al. 2021). WSPM exposure is often unavoidable and has been associated with the causation and exacerbation of respiratory diseases such as asthma, acute respiratory distress syndrome (ARDS), and chronic obstructive pulmonary disease (COPD) (Brunekreef and Holgate 2002; Zahran et al. 2018; Engels et al. 2024). Therefore, understanding the underlying mechanisms that control WSPM-induced lung injury and disease-related outcomes is critical.

It has been repeatedly shown that exposure to air pollutants induces cytochrome 450 (CYP) enzymes including CYP1A1, CYP1A2, and CYP1B1, in part via activation of the aryl hydrocarbon receptor (AHR) (Stading et al. 2021; Grimm et al. 2023). CYP enzymes are a superfamily of heme-containing monooxygenases that play essential and variable roles in xenobiotic and endogenous substrate metabolism. Although many studies have demonstrated a relationship between CYP enzymes and effects of environmental pollutants (John et al. 2010; Jaligama et al. 2018; Friberg et al. 2023), the consequences of CYP1B1 induction in airway epithelial cells (AECs) remain unclear, in part because it is difficult to untangle specific roles among the many overlapping roles and effects of other co-expressed CYPs.

CYP1B1 is unique that it shows only 41% and 40% amino acid sequence identity with human CYP1A1 and CYP1A2, respectively (Dutour and Poirier 2017). In addition, CYP1B1 is expressed primarily in extrahepatic tissues including the lungs. CYP1A1 is not constitutively expressed in any tissue but is inducible in all tissues, whereas CYP1A2 is primarily expressed in the liver (Buters et al. 1999; Grimm et al. 2023). Recent studies have identified roles for CYP1B1 in metabolism, inflammation, angiogenesis, pain, and cancer (Li et al. 2017; D’Uva et al. 2018; Chen et al. 2023; Sun et al. 2023). Also, CYP1A1 may attenuate hyperoxic lung injury, whereas CYP1B1 may contribute to injury (Lingappan et al. 2017; Veith et al. 2018; Stading et al. 2021). These findings suggest that CYP1B1 has context-dependent functionality and purpose.

In the present study, the expression of CYP1B1 in human bronchial epithelial cells (HBEC3-KT and BEAS-2B) was upregulated by treating cells with pine wood-derived WSPM. To dissect how CYP1B1 influences WSPM-induced AEC injury, we developed a CYP1B1 overexpressing BEAS-2B cell line and used a CYP1B1 inhibitor, TMS (2,3′,4,5′-Tetramethoxystilbene), as well as *Cyp1b1*-null mice in parallel studies of cell injury/repair and inflammatory signaling. We hypothesized that CYP1B1 would attenuate AEC/lung injury associated with WSPM exposure.

## Materials and methods

### Chemicals

2,3′,4,5′-Tetramethoxystilbene (TMS) was purchased from Cayman Chemical (Ann Arbor, MI). Pine WSPM was prepared as previously described (Deering-Rice et al. 2018). Dry Austrian pine wood from a tree growing in the Salt Lake Valley was burned using a laboratory pipe furnace at 750°C. Size-fractionated WSPM was collected using an Anderson cascade impactor (ThermoAndersen, Smyrna, Georgia), and fractions 5 to 7 (0.43 to 2.1 µm) were solubilized in DMSO at an initial concentration of 115 mg/ml for in vitro experiments. Pine WSPM was diluted into media as needed, with DMSO content ≤0.1%. The potency for each WSPM batch was verified using quantitative calcium flux assays for TRPA1 and TRPV3 activation, as described (Nguyen et al. 2020), and measuring the induction of CYP1B1 and TRPV3 transcripts as downstream markers of exposure. Criteria were: TRPA1 activation (>50% calcium flux relative to AITC at 100 µM); TRPV3 activation (>20% calcium flux relative to drofenine at 125 µM); TRPV3 mRNA induction (>5-fold control at 24 h after WSPM treatment at 10 µg/cm^2^); and CYP1B1 mRNA induction (>2-fold control at 24 h after WSPM treatment at 10 µg/cm^2^).

### Cell culture

HBEC3-KT cells (normal HBECs immortalized with CDK4 and hTERT) were purchased from ATCC (Manassas, VA) and cultured in Airway Epithelial Basal Medium supplemented with Bronchial Epithelial Cell Growth Kit (ATCC, Manassas, VA). Human bronchial epithelial cells (BEAS-2B) (ATCC, Manassas, VA) were grown in LHC-9. Cells were cultured in a humidified incubator at 37°C in a 95% air and 5% CO_2_ atmosphere. HBEC3-KT and BEAS-2B cells were passaged at approximately 80% confluence using trypsin (Gibco, TrypLE Express enzyme 1X), neutralized with complete growth medium, centrifuged, and replated at the desired density.

### CYP1B1-OE cells

The CYP1B1 open reading frame (ORF) was amplified from BEAS-2B cells treated with pine WSPM, using Platinum SuperFi PCR Master Mix (Thermo Fisher, Waltham, MA) and oligonucleotides A, 5′-CACCATGGGCACCAGCCTCAG-3′ (forward primer) and B, 5′-TTGGCAAGTTTCCTTGGCTTG-3′ (reverse primer). The amplified product was then cloned into the pcDNA3.1 Directional TOPO mammalian expression vector (Thermo Fisher, Waltham, MA) according to the manufacturer’s instructions followed by transformation of One Shot TOP10 chemically competent *E. coli* cells for amplification. Plasmid DNA was isolated using the GenElute HP Plasmid Miniprep Kit (Millipore Sigma) and the insert sequence and orientation were verified by sequencing. Oligonucleotide B was designed without a stop codon to fuse CYP1B1 in frame with the C-terminal V5 epitope and 6xHis tag included in the expression vector. CYP1B1-V5/His6 was then subcloned into the pcDNA5/FRT vector by PCR amplification (Platinum SuperFi PCR Master Mix) and oligonucleotides A (forward primer) and B (reverse primer) (Primer information is detailed in Table S1). The product was then purified using the QIAquick PCR purification kit (Qiagen, Germantown, MD) and cloned into the pcDNA5/FRT vector using Kpn I and BamH I restriction enzymes and ligation (Thermo Fisher, Waltham, MA). Sequencing verified plasmid DNA was then used to create the CYP1B1 overexpressing BEAS-2B cell lines. The host Flp-In BEAS-2B cell line with a single FRT integration site was created as previously described (Almestica-Roberts et al. 2024). Human CYP1B1 over-expressing cells were created by co-transfecting the Flp-In BEAS-2B host cell line with the pOG44 Flp recombinase expression vector and the pcDNA5/FRT vector harboring CYP1B1-V5/His using FuGene6 transfection reagent (Promega, Madison, WI) according to the manufacturer’s instructions. The CYP1B1 over-expressing cell lines were selected for hygromycin resistance (20 µg/mL) and maintained in LHC-9 media containing hygromycin.

### Pine WSPM treatment

Treatments were normalized to surface area (µg/cm^2^). Stock WSPM suspensions were prepared at 115 mg/mL in DMSO and diluted in culture media to achieve the desired surface-area-normalized dose. Cells were treated using the following volumes: 150 µL per well for 96-well plates, 1 mL per well for 12-well plates, and 2 mL per well for 6-well plates. The corresponding surface areas are standard manufacturer specifications (0.32 cm^2^ per 96-well, 3.8 cm^2^ per 12-well, and 9.5 cm^2^ per 6-well).

### Cytotoxicity assays

Cells were cultured in a 96-well plate at a density of 25,000 cells per well for 24 h. Cells were then treated with pine WSPM suspended in culture media at various concentrations to yield treatment area doses of 1 to 50 µg/cm^2^ and incubated for an additional 24 h. After treatment, residual cell viability was measured using the Dojindo Cell Counting Kit-8 (Dojindo, Rockville, MD) according to the manufacturer’s protocol.

### Proliferation and kinetic scratch wound assays

To quantify proliferation rates, cells were plated at 25,000 cells/cm^2^ with or without treatments in an Image-Lock 96-well culture plate (Essen BioSciences, Ann Arbor, MI). Images were captured for 84 h at 1-h intervals using an IncuCyte ZOOM real-time live-cell imaging system at 10× magnification. The 84-h duration was selected to allow sufficient time for cumulative differences in proliferation to emerge between groups, as changes in growth kinetics typically manifested gradually.

For scratch wound assays, cells were plated and grown for 48 h to 100% confluence in an Image-Lock 96-well culture plate. Precision mechanical scratch wounds were made using the Incucyte WoundMaker 96-pin wound-making tool (Essen BioSciences). The cells were then washed 3× with PBS to remove the detached cells, and then the desired treatments were added. Serial scratch wound closure images were captured using the IncuCyte ZOOM system at 10× magnification. Scratch assays were limited to 48 h because epithelial wound closure occurred rapidly, and extending the assay beyond this timeframe resulted in near-complete closure, hindering discrimination of migration differences between groups. Wound closure was measured as the percentage of the initial gap covered by migrating cells. Analysis parameters were set and the image series was analyzed using IncuCyte ZOOM software, as reported in previous study (Burrell et al. 2021). Cell images were labeled with yellow masks using Cellpose 2.0 as described previously (Memon et al. 2023).

### Immunocytochemical analysis of NRF2 in HBEC3-KT cells

Cells were cultured in a 24-well plate at a density of 25,000 cells per well for 3 days. Then, cells were treated with pine WSPM and/or TMS in culture media for 24 h. After treatment, cells were then fixed with 4% paraformaldehyde for 30 min and permeabilized with 0.2% Triton X-100 for 20 min at room temperature in the dark. Nonspecific antibody binding was blocked by incubating cells in 10% normal goat serum for 1 h at room temperature. Cells were then incubated with a rabbit monoclonal primary antibody for NRF2 (1:1,000, ab62352; Abcam) overnight at 4°C, followed by incubation with a goat-anti-rabbit secondary antibody conjugated with Alexa-Fluor488 (1:500, A-11001; Invitrogen). After incubation with the antibodies, cell nuclei were stained with Hoechst 33342, and cells were postfixed with 4% paraformaldehyde for 20 min. The plates were then imaged on ImageXpress PICO at 40× magnification (Molecular Devices, San Jose, CA).

### Mice

Experimental protocols were approved by the University of Utah Institutional Animal Care and Use Committee. C57BL/6J mice (20 to 25 g; 6 to 8 wk) were obtained from Jackson Laboratories. *Cyp1b1^−/−^* mice (*C57BL/6J* background) were obtained from Dr Bhagavatula Moorthy of the Baylor College of Medicine (Houston, Texas), under a material transfer agreement with Dr Frank J. Gonzalez, of the National Cancer Institute (Bathesda, MD). *Cyp1b1^−/−^* mice were maintained as a breeding colony on site. All mice were housed in an AAALAC-approved vivarium under controlled environmental conditions consisting of 12-h light/12-h dark cycles, at a temperature of 23°C to 26°C, and 40% to 50% relative humidity. The mice were fed standard lab chow and water ad libitum. Mice were genotyped by PCR using genomic DNA obtained from ear punches. The primer pairs used to distinguish the wild-type and knockout alleles are shown in Table S2, as described previously (Castro et al. 2008).

The age-matched wild-type and *Cyp1b* knockout mice were treated with freshly prepared pine WSPM (resuspended daily in DMSO) at a dose of 0.5 mg/kg (12.5 μg total PM per dose) or saline every other day via the oropharyngeal route, as previously described (Deering-Rice et al. 2018). 24 h after the third exposure, bronchoalveolar lavage fluid (BAL) was collected, subjected to cytospin, and stained with Wright-Giemsa stain to quantify cell abundances, as previously described (Cowley et al. 2025). Cytospin images were acquired using an EVOS FL Auto microscope (ThermoFisher, Waltham, MA) at 20× magnification. Following BALF collection, the lungs were perfused with saline via a hole at right ventricle. The right main bronchus was ligated with a 3-0 silk suture, and the right lung lobes were collected into RNALater and stored at 4°C for subsequent RNA isolation. The left lung lobe was then inflated with 10% neutral-buffered formalin and fixed in a 10% neutral-buffered formalin for 48 h. The fixed tissue was rinsed with 2% sucrose and dehydrated in 70% ethanol, as previously described (Cowley et al. 2025). Lungs were embedded in paraffin, and serially sectioned (5 μm) followed by staining with Hematoxylin and Eosin by ARUP Laboratories (Salt Lake City, UT). The H&E images were acquired using an ImageXpress Pico automated cell imaging system at 20× magnification.

### Quantitative real-time polymerase chain reaction assays

For lung tissue mRNA analysis, the left lung lobe, preserved in RNALater at 4°C, was homogenized in Trizol Reagent (1 mL/50 to 100 mg of tissue), and phase separated with chloroform. The RNA was then precipitated with ethanol and subsequently purified using the PureLink RNA Mini Kit (Invitrogen, Carlsbad, CA), as described (Cowley et al. 2025).

For cellular mRNA expression analyses, cells were plated at a density of 25,000 to 30,000 cells per cm^2^ and cultured for 72 h prior to treatment. Following treatment, the culture media was removed, and the plates were stored at −80°C until RNA purification. Total RNA from cultured cells was isolated using the PureLink RNA Mini Kit (Invitrogen, Carlsbad, CA).

In all cases, RNA concentration and purity were measured spectrophotometrically using the NanoDrop One Microvolume UV-Vis Spectrophotometer (Thermo Scientific, Waltham, MA). cDNA was synthesized from total RNA using the ABI High-Capacity cDNA Synthesis Kit with RNase inhibitor (Applied Biosystems, Foster City, CA), according to previously published methods (Sun et al. 2025).

Taqman probe-based qPCR assays were performed using a Life Technologies QuantStudio 6 Flex instrument. The ΔΔC_T_ method was used to calculate target gene expression by normalizing to β2-microglobulin (β2M; Hs00187842_m1) for human samples or glyceraldehyde 3-phosphate dehydrogenase (Gapdh; Mm99999915_g1) for mouse samples. Fluorescent probes and primers to distinguish the spliced (XBP1s) and unspliced (XBP1u) transcripts of X-box binding protein-1 (XBP1) were same as previously described (Maiuolo et al. 2011; Nguyen et al. 2020). Probe details are listed in Table S3.

### Mouse tracheal epithelial cell isolation and culture

Wild-type C57BL/6 mice and *Cyp1b1^−/−^* mice were used to isolate mouse tracheal epithelial cell (MTECs) as described previously (Eenjes et al. 2018). Briefly, tracheas were collected and stored in tubes on ice in solution 1 (Ham’s F12 media supplemented with 100 units/ml penicillin and 100 µg/ml streptomycin). Then, the esophagus and other debris were removed from the tracheas and which were then cut lengthwise. The tissue was then placed in solution 1 containing 150 µg/ml pronase and incubated at 4°C overnight. After an additional 20-min incubation at room temperature, the tubes were gently inverted 15 times. To stop the digestion, 10% FBS was added, and the media was transferred to a 50 mL tube. The tracheas were subsequently washed three times with 7 mL Ham’s F12 media supplemented with 10% FBS, 100 units/ml penicillin, and 100 µg/ml streptomycin. During each wash, the tubes were inverted 5 times, and the media was collected into the same 50 mL tube. The cell suspension was then centrifuged at 400 ×*g* at 4°C for 10 min. The cell pellet was resuspended in 2 mL DNase solution (Ham’s F12 media supplemented with 100 units/ml penicillin, 100 µg/ml streptomycin, 0.5 mg/mL crude pancreatic DNase I, 1 mg/mL BSA) and incubated on ice for 5 min before another centrifugation at 400 ×*g* at 4°C for 10 min. After removing the DNase solution, the cell pellet was resuspended in 15 mL KSFM growth media (KSFM containing 100 units/ml penicillin and 100 µg/ml streptomycin, 0.025 µg/ml Murine EGF, 0.03 mg/ml Bovine Pituitary Extract, 1 µM isoproterenol, and freshly added Y-27632 and DAPT). The cells were then cultured for further expansion and testing.

### Statistical analysis

Results are presented as the mean ± SD from a minimum of 3 biological replicates, as detailed in figure legends. Differences between groups were analyzed using a two-tailed Student’s *t*-test, one-way ANOVA, or two-way ANOVA with a Bonferroni or other appropriate post hoc test at the 95% confidence level. All statistical analyses were performed using GraphPad Prism 10.0.2 (GraphPad Software Inc., La Jolla, CA). Comprehensive *P*-value results for all figures are summarized in Table S4.

## Results

### CYP1B1 mRNA expression was induced in AECs by pine WSPM treatment

**Fig. 1.**
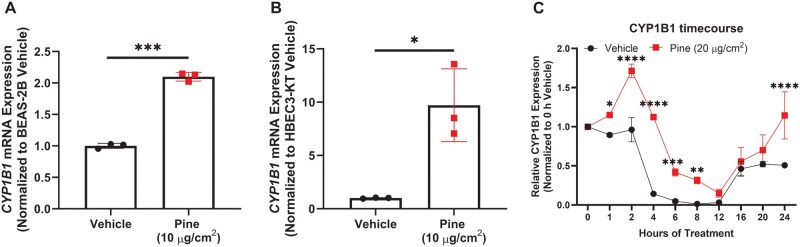
CYP1B1 mRNA was upregulated in both (A) BEAS-2B and (B) HBEC3-KT cell lines treated with pine WSPM (10 µg/cm^2^) for 24 h. Data were normalized to control B2B FRT or HBEC3-KT cells and are the mean±SD (n = 3). Statistical testing was performed using a two-tailed Student’s *t*-test.^*^P < 0.05, ^***^P < 0.001. (C) Temporal changes in *CYP1B1* mRNA in HBEC3-KT cells after pine WSPM treatment (20 µg/cm^2^). Each time point was normalized to 0-h vehicle-treated HBEC3-KT cells and is presented as the mean±SD (n = 3). Statistical testing was performed using two-way ANOVA with Bonferroni’s multiple comparisons test. ^*^P < 0.05, ^**^P < 0.01, ^***^P < 0.001, ^****^P < 0.0001.

It is widely reported that CYP1B1 is induced by environmental pollutants (e.g., 2,3,7,8-tetrachlorodibenzo-*p*-dioxin; TCDD) (Sutter et al. 1994; Buters et al. 1999). Here, *CYP1B1 mRNA* was induced in both BEAS-2B and HBEC3-KT AECs following exposure to pine WSPM for 24 h at a concentration of 10 µg/cm^2^ (Fig. 1A and B). The kinetics of CYP1B1 mRNA induction were further explored in HBEC3-KT cells treated with WSPM (20 µg/cm^2^). Cyclical changes expression was observed in both control and treated cells; initially increasing, then decreasing, then increasing again, with generally higher levels of expression for WSPM-treated cells (Fig. 1C). The transient reduction in CYP1B1 expression observed between 4 and 12 h likely reflects global suppression of transcription associated with endoplasmic reticulum stress (ERS) and/or an early culture-associated adaptive response. Alternatively, transcripts for transient receptor potential vanilloid-3 (TRPV3), which we have previously identified as a WSPM-induced gene (Nguyen et al. 2020), demonstrated a rapid and sustained increase over the 24-h treatment period (Fig. S1), consistent with its reported role in attenuating ERS.

### CYP1B1 modulated the cytotoxic effects of WSPM in AECs

**Fig. 2.**
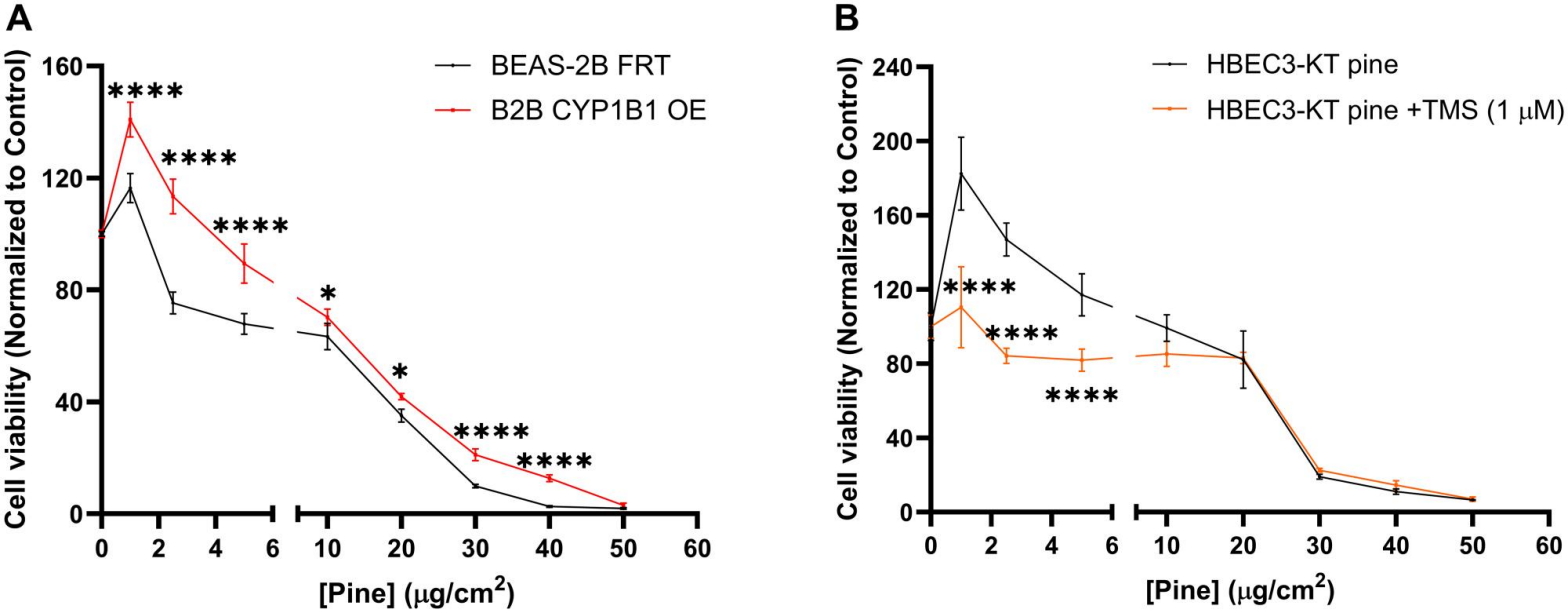
(A) CYP1B1 overexpression in BEAS-2B cells reduced cytotoxicity after 24-h treatment with increasing concentrations of pine WSPM. (B) The CYP1B1 inhibitor TMS increased cytotoxicity in HEBC3-KT cells after 24-h pine WSPM treatment. Data were normalized to vehicle-treated controls and is presented as the mean±SD (n = 3). Statistical testing was performed using two-way ANOVA with Bonferroni’s multiple comparisons test. In (A), comparisons were made between B2B CYP1B1 overexpressing and B2B FRT cells at each WSPM concentration; in (B), comparisons were made between pine-TMS-treated and pine-treated cells at each concentration. ^*^P < 0.05, ^****^P < 0.0001.

Basal *CYP1B1* mRNA expression varies among human lung cell types (Fig. S2A). To further explore the implications of induction and variable CYP1B1 expression in the context of WSPM injury, we generated BEAS-2B cells that stably overexpress CYP1B1 (B2B CYP1B1OE cells) and used the CYP1B1 inhibitor TMS. The relative expression of CYP1B1 protein in BEAS-2B FRT (B2B FRT) and B2B CYP1B1OE cells is shown in Fig. S2B. WSPM treatment of BEAS-2B FRT host cells for 24 h caused a dose-dependent decrease in cell viability, which was significantly attenuated by CYP1B1 overexpression across the 1 to 40 µg/cm^2^ concentration range of WSPM (*P* < 0.05) (Fig. 2A). Alternatively, TMS cotreatment of HBEC3-KT AECs markedly increased cytotoxicity at WSPM concentrations below 5 µg/cm^2^ (*P* < 0.0001) (Fig. 2B).

### CYP1B1 modulated AEC proliferation and migration following WSPM treatment

**Fig. 3.**
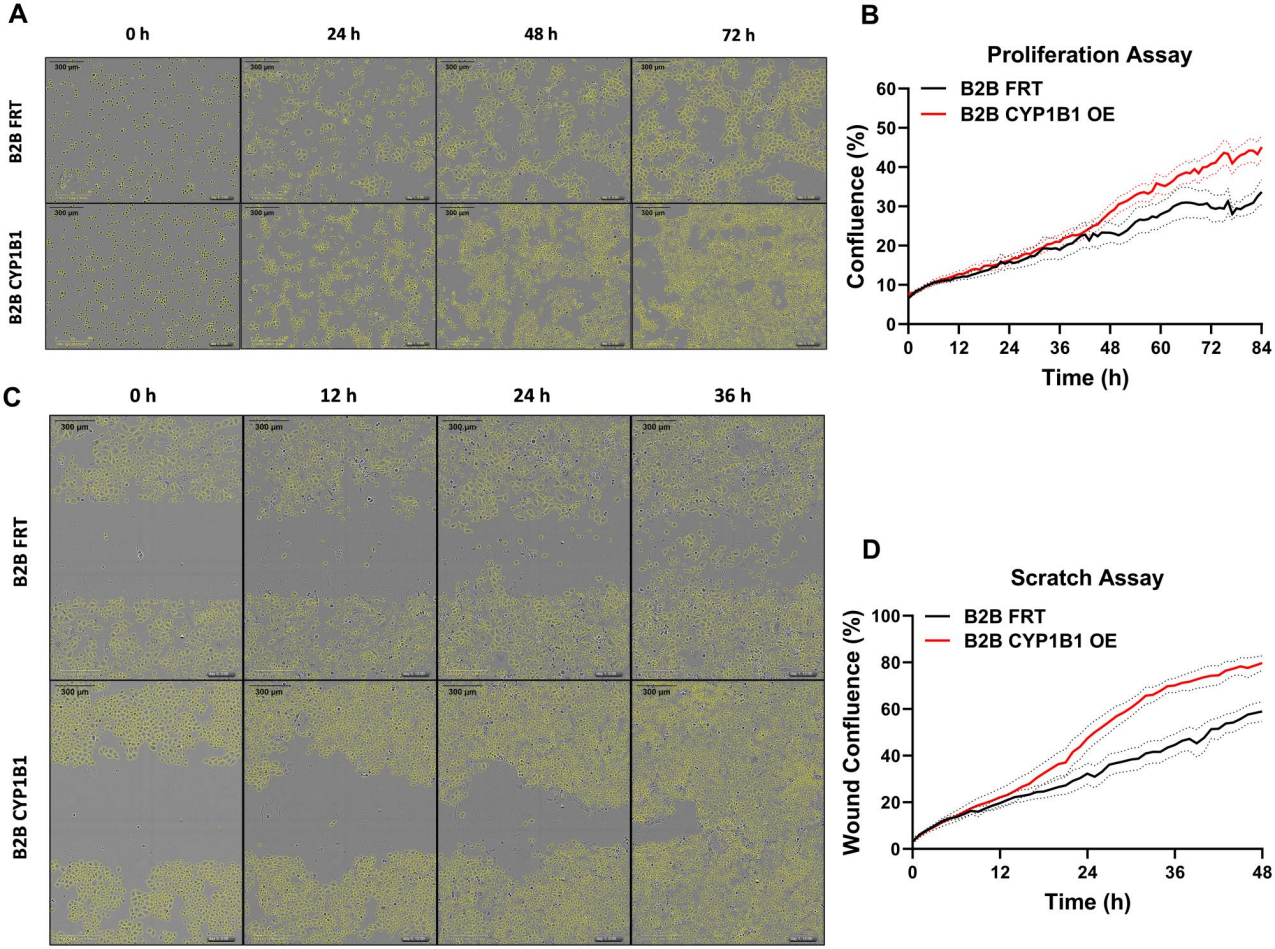
Stable overexpression of CYP1B1 in BEAS-2B cells promoted (A and B) cell proliferation and (C and D) scratch wound gap closure. Live-cell microscopy images were acquired using an IncuCyte ZOOM real-time live-cell imaging system at 10× magnification. Cell proliferation and scratch wound closure were monitored over time, and representative images are shown.

**Fig. 4.**
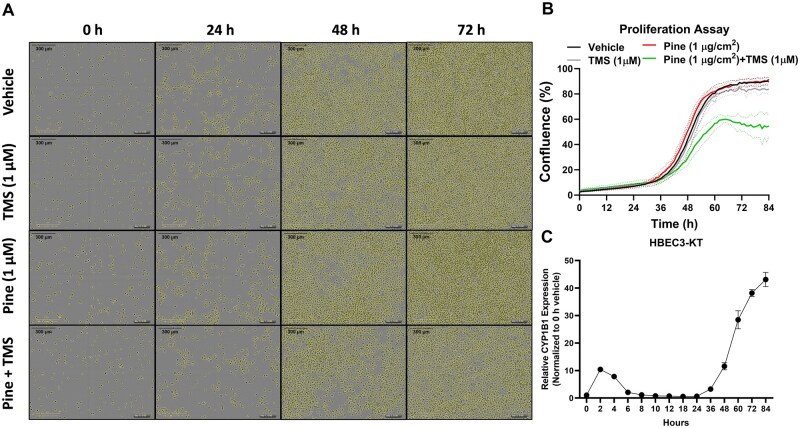
(A and B) TMS, a potent CYP1B1 inhibitor, suppressed cell proliferation/monolayer formation by HBEC3-KT cells treated with pine WSPM (1 µg/cm^2^). (C) CYP1B1 mRNA was rapidly up-regulated after 36 h of plating and remained elevated up to 84 h in normal HBEC3-KT cells. Live-cell microscopy images were acquired using an IncuCyte ZOOM real-time live-cell imaging system at 10× magnification. Cell proliferation was monitored over time, and representative images are shown.

Previous research suggested that CYP1B1 fosters cell proliferation, migration, and invasion in MCF-7 and MCF-10A cells (Kwon et al. 2016). Representative live-cell microscopy images illustrating monolayer formation and scratch wound gap closure as a function of CYP1B1 expression in BEAS-2B cells are shown in Fig. 3A and C, with quantification in Fig. 3B and D. Cell masks (yellow) were applied to highlight the differences in proliferation and scratch wound closure using Cellpose 2.0 as described previously (Memon et al. 2023). Notably, CYP1B1 overexpression accelerated epithelial cell proliferation and migration, resulting in more rapid monolayer formation and scratch wound gap closure in vitro. Alternatively, inhibiting CYP1B1 using TMS significantly reduced proliferation/monolayer formation among HBEC3-KT cells treated with WSPM (1 µg/cm^2^; Fig. 4A and B). Divergence in monolayer formation was observed after ∼36 h of treatment, around the same time that CYP1B1 mRNA levels increased in HBEC3-KT cells (Fig. 4C), highlighting the potential association between CYP1B1 expression, cell proliferation, and migratory responses following injury.

### CYP1B1 influenced ERS induced by WSPM

**Fig. 5.**
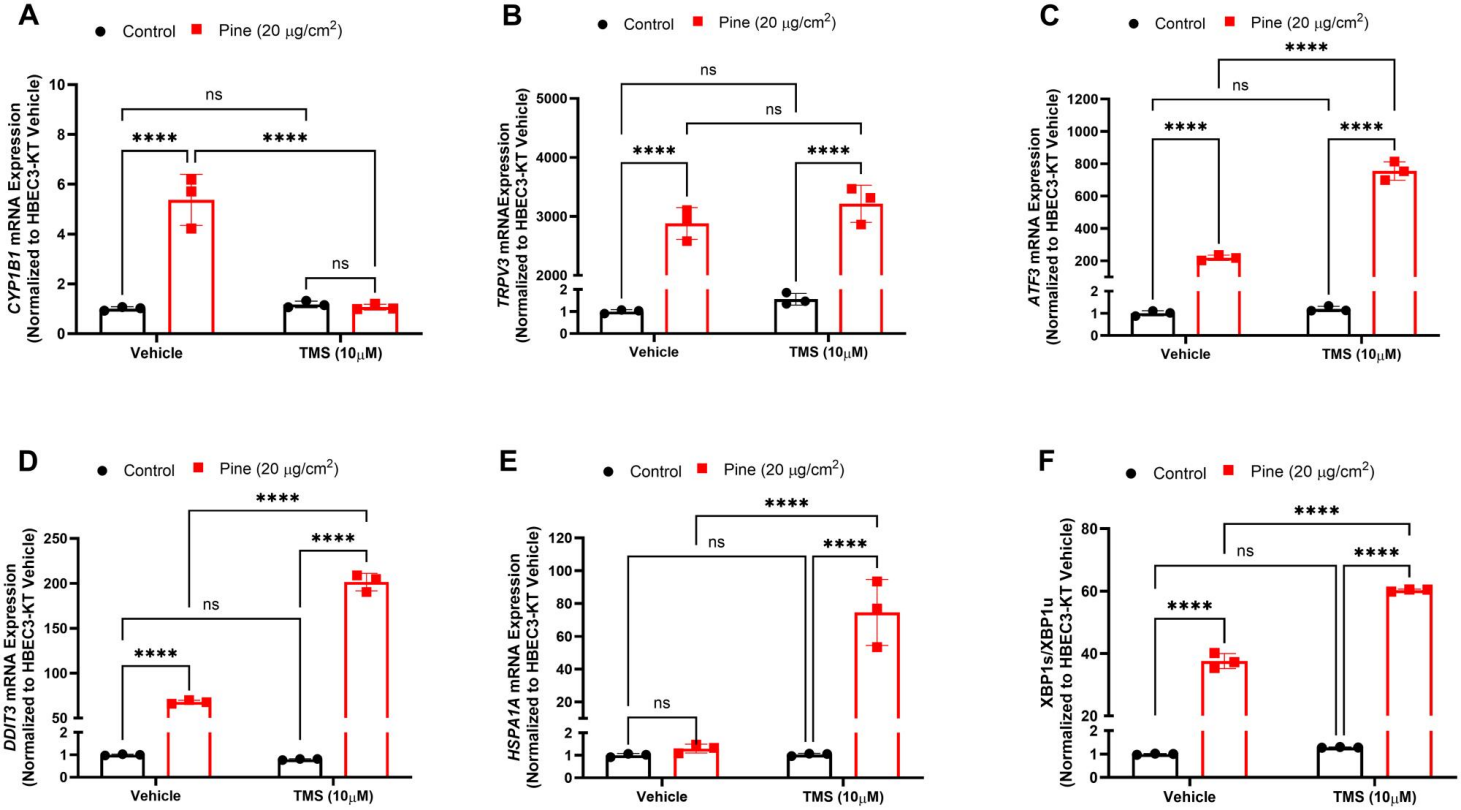
(A) TMS inhibited CYP1B1 induction in WSPM-treated HBEC3-KT cells and (B) had no effect on *TRPV3* induction, a biomarker of ERS. (C–F) TMS exacerbated the induction of other prototypical ERS biomarkers including *ATF3*, *DDIT3*, *HSPA1A*, and *XBP1* mRNA splicing, indicating a more robust response. Data were normalized to control HBEC3-KT cells and presented as the mean±SD (n = 3). Statistical testing was performed using two-way ANOVA with Tukey’s multiple comparisons test. ^****^P < 0.0001.

The influence of CYP1B1 on WSPM-induced ERS in HBEC3-KT cells was assessed based on a prior study indicating ERS as a mechanism of WSPM toxicity in AECs (Nguyen et al. 2020). Surprisingly, TMS suppressed *CYP1B1* mRNA induction by WSPM (Fig. 5A) while having no effect on *TRPV3* expression (Fig. 5B). Further, inhibiting CYP1B1 exacerbated the expression of multiple mRNA biomarkers of ERS including the PERK/eIF2αK3 pathway markers *ATF3* (∼3.5-fold greater than WSPM control) and *DDIT3* (∼3-fold greater than WSPM control), the chaperone *HSPA1A* (∼57-fold greater than WSPM control), and the IRE1α/β biomarker spliced *XBP1* (∼1.6-fold greater than the WSPM control) (Fig. 5C–F).

ERS biomarker expressions in B2B FRT and B2B CYP1B1OE cells were also compared. As illustrated in Fig. S3A, *CYP1B1* mRNA was overexpressed ∼135-fold in B2B CYP1B1OE cells compared with B2B FRT cells, presumably related to CYP1B1 overexpression promoting proliferation/monolayer formation and wound gap closure vs. cell cycle arrest in nonoverexpressing cells or cells treated with TMS. Notably, *TRPV3* mRNA expression was also elevated (∼4-fold) in CYP1B1OE cells relative to BEAS-2B FRT cells ∼16-fold after treatment with WSPM (Fig. S3B). Finally, B2B CYP1B1OE cells also exhibited slightly increased levels of *DDIT3*, *HSPA1A*, and *XBP1* splicing (Fig. S3C–F), albeit less than in HBEC3-KT cells, again suggesting a role for CYP1B1 in fettering ERS and a potential shift toward stress adaptation involving TRPV3 induction.

### CYP1B1 inhibited NRF2 in AECs

**Fig. 6.**
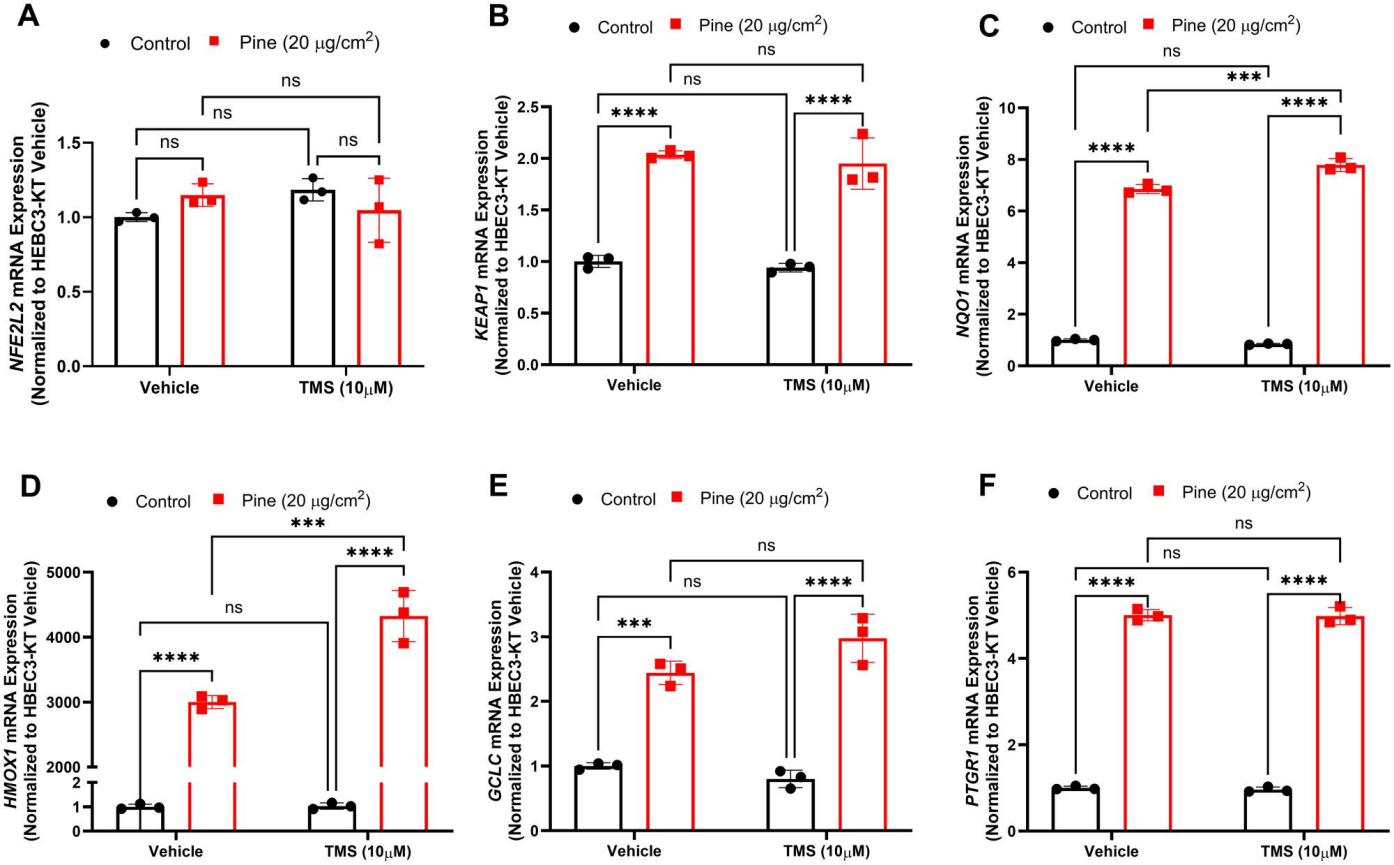
Inhibition of CYP1B1 had no effect on (A) *NFE2L2* or (B) *KEAP1* mRNA expression but increased the expression of mRNA for common NRF2 target genes: (C) *NQO1*, and (D) *HMOX1* in HBEC3-KT cells treated with WSPM (20 µg/cm^2^; 24 h). (E) *GCLC* and (F) *PTGR1* expressions were also not impacted. Data were normalized to control HBEC3-KT cells and presented as the mean±SD (n = 3). Statistical testing was performed using two-way ANOVA with Tukey’s multiple comparisons test. ^***^P < 0.001, ^****^P < 0.0001.

ERS, oxidative stress, and NRF signaling are integrated (Zhang et al. 2019, 2023) with evidence that PERK/EIF2αK3 and IRE1α/β activate NRF2 (Cullinan and Diehl 2004), and that NRF2 controls ERS (Li et al. 2020; Naresh Amin et al. 2021). The KEAP1-NRF2 pathway regulates cytoprotective responses to oxidative stress by modulating the expression of NAD(P)H dehydrogenase (quinone 1) (NQO1), heme-oxygenase (HO)-1 (HMOX1), glutamate-cysteine ligase catalytic subunit (GCLC), prostaglandin reductase-1 (PTGR1) (Li et al. 2022). Here WSPM treatment induced the expression of *KEAP1*, *NQO1*, *HMOX1*, *GCLC*, and *PTGR1*, but not *NFE2L2* mRNA (Fig. 6). Further, inhibition of CYP1B1 with TMS enhanced the induction of *NQO1* and *HMOX1* by WSPM (Fig. 6C and D).

**Fig. 7.**
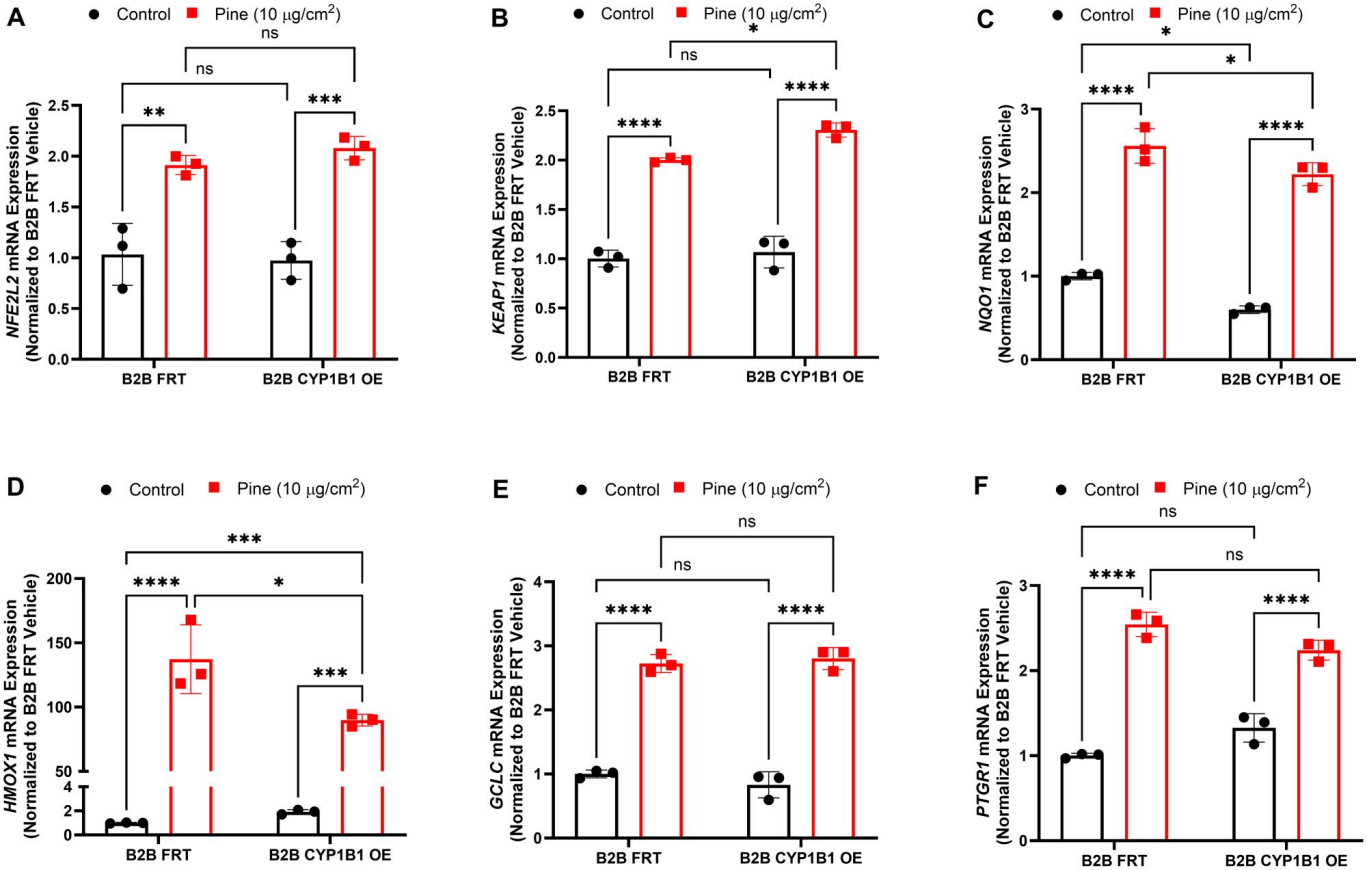
CYP1B1 overexpression had no effect on (A) *NFE2L2* or (B) *KEAP1* mRNA expression but downregulated and attenuated the induction of mRNA for (C) *NQO1* and (D) HMOX1 in BEAS-2B cells treated for 24 h with pine WSPM (20 µg/cm^2^). (E) *GCLC* and (F) *PTGR1* expressions were also not impacted. Data were normalized to control B2B FRT cells and shown as the mean±SD (n = 3). Statistical testing was performed using two-way ANOVA with Tukey’s multiple comparisons test. ^*^P < 0.05, ^**^P < 0.01, ^***^P < 0.001, ^****^P < 0.0001.

**Fig. 8.**
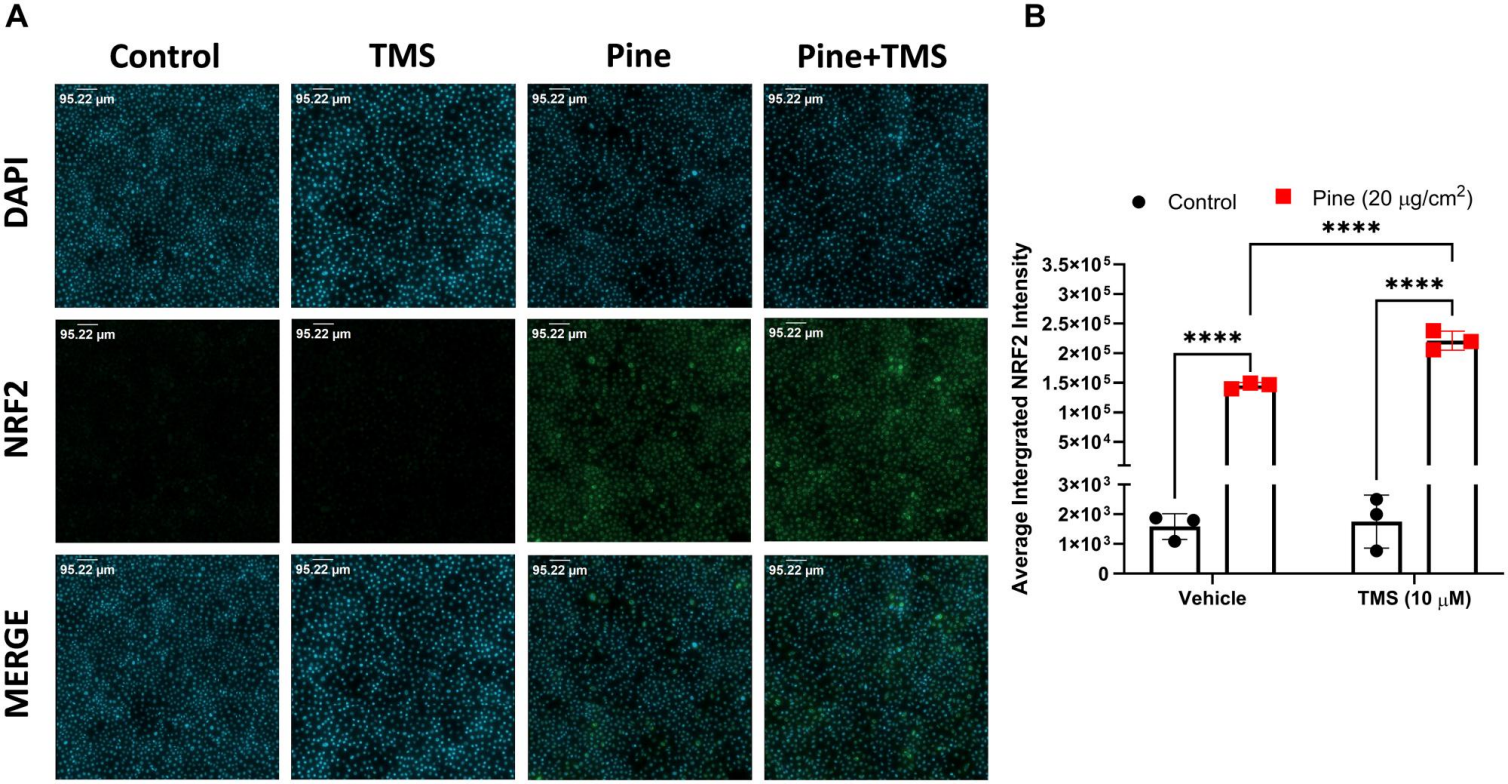
(A) Inhibition of CYP1B1 enhanced NRF2 abundance and nuclear translocation in HBEC3-KT cells exposed to WSPM for 24 h at 20 µg/cm^2^. Images were acquired using an ImageXpress Pico automated cell imaging system at 40× magnification. (B) Data were normalized to control HBEC3-KT cells and are shown as the mean±SD (n = 3). Statistical testing was performed using two-way ANOVA with Tukey’s multiple comparisons test. ^****^P < 0.0001.

*KEAP1*, *NQO1*, *HMOX1*, *GCLC*, *PTGR1* but not *NFE2L2* mRNA expression were also induced by WSPM in B2B FRT cells (Fig. 7). However, *KEAP1* mRNA expression increased, while *NQO1* and *HMOX1* mRNA decreased in B2B CYP1B1OE cells treated with WSPM (Fig. 7B–D); there was no significant difference in *GCLC* and *PTGR1* mRNA induction (Fig. 7E and F). Immunocytochemical analysis combined with gene expression data confirmed that WSPM treatment promoted NRF2 stability and nuclear translocation, and that the inhibition of CYP1B1 enhanced NRF2-driven gene expression in HBEC3-KT cells treated with WSPM (Fig. 8), consistent with the effects of TMS on *HMOX1* and *NQO1* induction.

### CYP1B1 impacted IL8 regulation by AECs

**Fig. 9.**
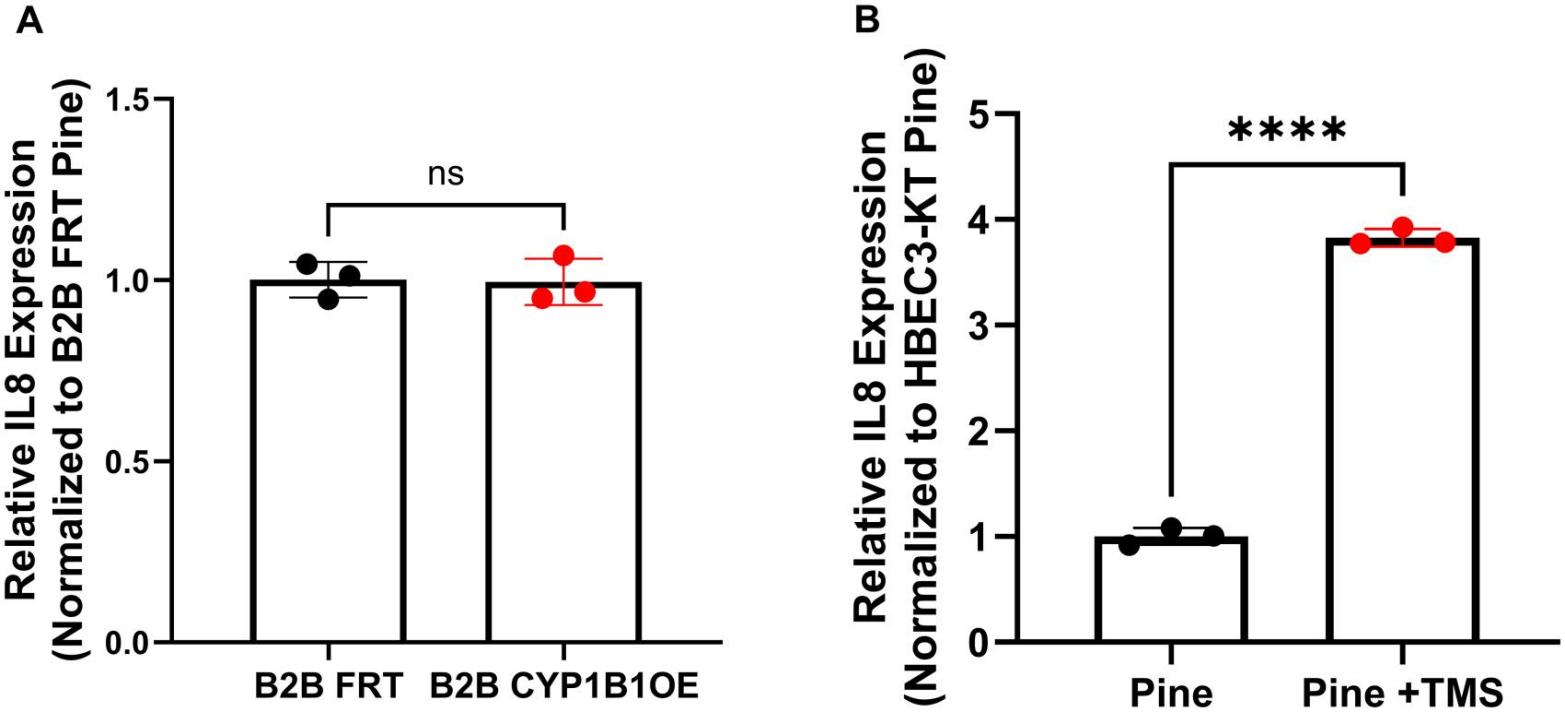
(A) Equivalent *IL8* mRNA expression in BEAS-2B FRT and BEAS-2B CYP1B1 OE cells treated with pine WSPM (10 µg/cm^2^) for 24 h. (B) Exacerbated *IL8* mRNA expression in HBEC3-KT cells treated with TMS (10 µM) and pine WSPM (20 µg/cm^2^) for 24 h. Data were normalized to B2B FRT or HBEC3-KT cells treated with pine and are shown as the mean±SD (n = 3). Statistical testing was performed using two-tailed Student’s *t*-test. ^****^P < 0.0001.

ERS and the NRF2 pathway also influence the expression of the proinflammatory chemokine IL8 (Zhang et al. 2005; Vij et al. 2006, 2008; Conciatori et al. 2020). To further investigate the role of CYP1B1 in modulating inflammatory responses to WSPM, we examined *IL8* mRNA expression in AECs under conditions of CYP1B1 overexpression and inhibition. Overexpression of CYP1B1 in B2B FRT cells did not significantly alter *IL8* mRNA expression compared with B2B FRT cells when exposed to WSPM, suggesting that increased CYP1B1 abundance alone is insufficient to suppress *IL8* transcription in the context of WSPM exposure (Fig. 9A). In contrast, inhibition of CYP1B1 activity by TMS in HBEC3-KT cells markedly increased *IL8* mRNA expression following WSPM treatment (∼4-fold greater than WSPM control) (Fig. 9B). This pronounced induction of *IL8* upon CYP1B1 inhibition indicates that CYP1B1 exerts a repressive effect on *IL8* transcription in response to WSPM, suggesting that CYP1B1 may modulate inflammation, in part through regulation of ERS and NRF2 signaling.

### Cyp1b1 deficiency promoted lung inflammation and Cyp1a1 mRNA expression in mice

**Fig. 10.**
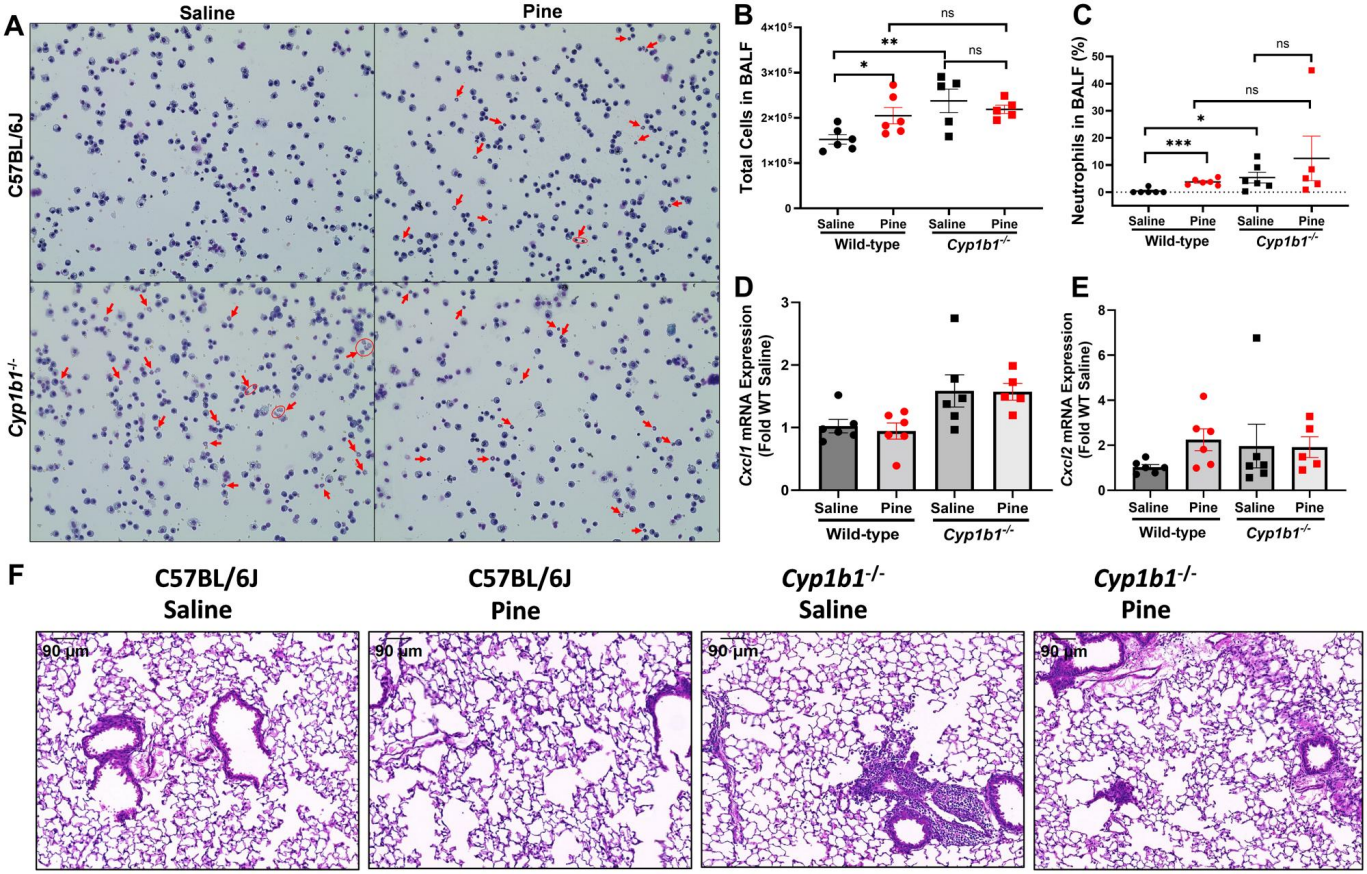
*Cyp1b1* deficiency promoted lung inflammation independent of WSPM. (A) Representative images (20×) of cells recovered in BALF of wild-type C57Bl/6J and *Cyp1b1*-deficient mice treated with saline or pine WSPM. Red arrows correspond to neutrophils. Expanded images of representative cells are shown in Fig. S5. (B and C) The total cells and neutrophils in BALF were significantly increased in *Cyp1b1*-deficient mice, which parallelled a slight induction of (D) *Cxcl1* but not (E) *Cxcl2* mRNA expression. Data were normalized to wild-type mice treated with saline and shown as the mean ± S.E.M. (n = 6). Statistical testing was performed using one-way ANOVA with Tukey’s multiple comparisons test. ^*^P < 0.05, ^**^P < 0.01, ^***^P < 0.001. (F) Representative H&E images of C57Bl/6J and *Cyp1b1*^*−*/*−*^ lungs treated with saline or WSPM using an ImageXpress Pico automated cell imaging system at 20× magnification.

Wild-type C57Bl/6J and *Cyp1b1*-deficient mice were treated with saline or WSPM every other day for a span of 6 days via the oropharyngeal route, as described in our previous study (Deering-Rice et al. 2018). While macrophages dominated BAL cells, the total number of cells and neutrophils recovered in the BAL of *Cyp1b1*-deficient mice was elevated regardless of treatment. Further, while the total number of cells and neutrophils in wild-type mice exposed to pine WSPM also increased relative to the saline control, no significant differences were observed in *Cyp1b1*-deficient mice (Fig. 10A–C). *Cxcl1* and *Cxcl2* (murine functional homologues of human IL8) mRNAs were also more abundant in *Cyp1b1^−/−^* mouse lung tissue in the absence or presence of pine WSPM (Fig. 10D and E). Histological examination of H&E-stained lungs also provided evidence of higher basal inflammation in the *Cyp1b1*-deficient mice, with less effect of WSPM treatment (Fig. 10F).

**Fig. 11.**
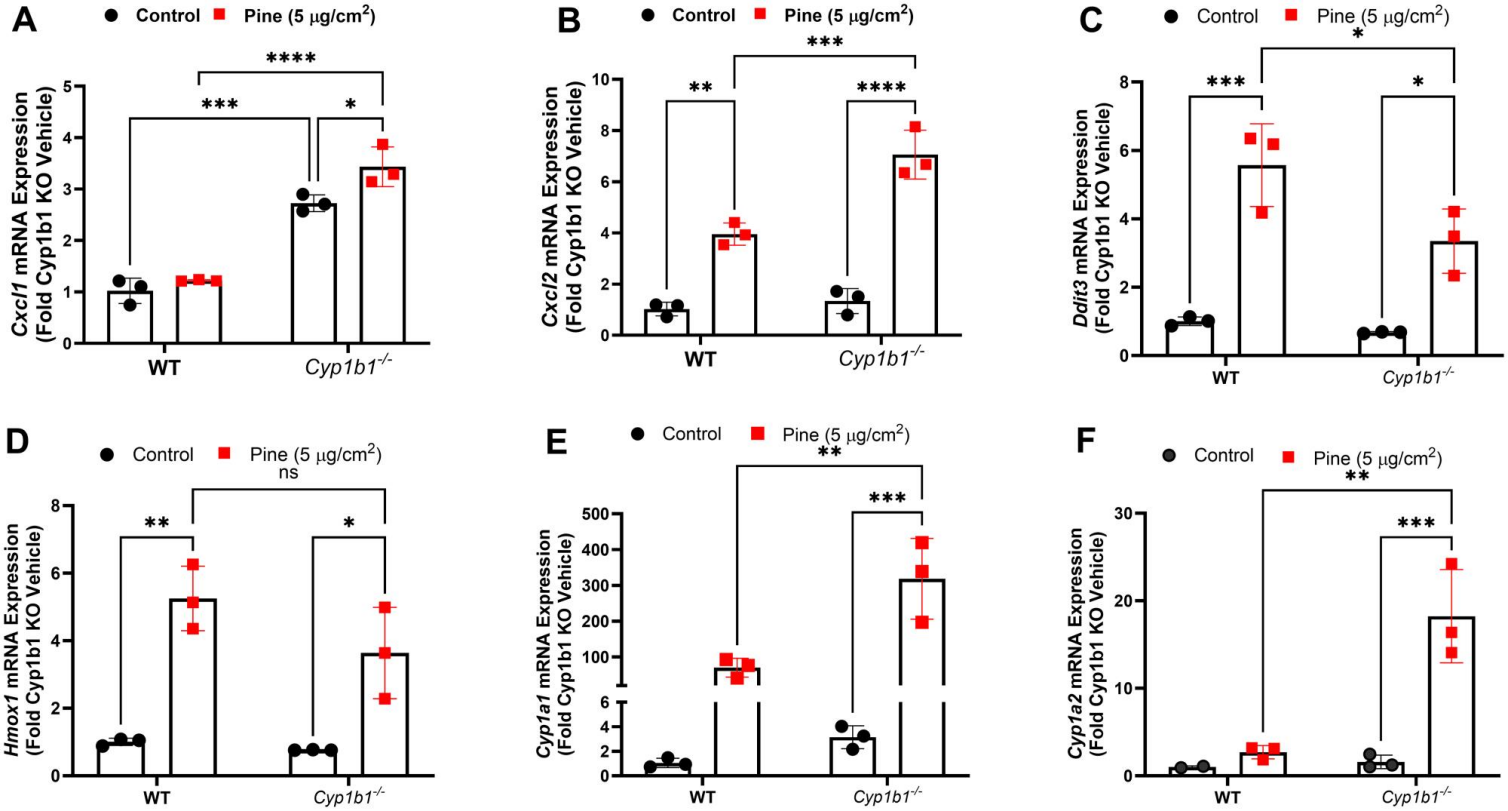
(A–F) Expression of *Cxcl1, Cxcl2*, *Ddit3*, *Hmox1*, *Cyp1a1*, and *Cyp1a2 mRNA* in wild-type and *Cyp1b1^−/−^* derived MTECs treated with pine WSPM (5 µg/cm^2^; 24 h). Data were normalized to control wild-type MTECs and shown as the mean±SD (n = 3). Statistical testing was performed using two-way ANOVA with Tukey’s multiple comparisons test. ^*^P < 0.05, ^**^P < 0.01, ^***^P < 0.001, ^****^P < 0.0001.

Intriguingly, *Cyp1a1* mRNA expression, but not that of *Cyp1a2*, *Cyp2j6*, or *Cyp2f2*, was also elevated in *Cyp1b1*-deficient mice with or without WSPM treatment (Fig. S4) suggesting compensatory upregulation *Cyp1a1* associated with *Cyp1b1*-deficiency. MTECs isolated from wild-type C57BL/6J or *Cyp1b1*-deficient mice were used to further link the human AEC in vitro and *mouse* experiments. *Cxcl1* (basally) and *Cxcl2* were induced after WSPM treatment to a greater degree in *Cyp1b1*-deficient cells (Fig. 11A and B). *Ddit3* but not *Hmox1* induction was suppressed, consistent with the effects of CYP1B1 overexpression in human AECs (Fig. 11C and D), presumably reflecting compensatory upregulation of *Cyp1a1* and *Cyp1a2* in *Cyp1b1^−/−^*-derived MTECs treated with pine (Fig. 11E and F), which were consistent with qPCR results from mouse lungs.

## Discussion

The purpose of this study was to determine the significance of CYP1B1 induction in response to WSPM-induced AEC injury in a broader effort to understand the role of CYP1B1 in AEC injury/repair. We found that CYP1B1 was induced by WSPM in human bronchial epithelial cells (HBEC3-KT and BEAS-2B) as well as in WT mice. We observed an attenuation of cytotoxicity associated with lower concentrations of WSPM treatment in B2B CYP1B1OE cells compared with B2B FRT cells, and an increase in HBCE3-KT cells cotreated with the CYP1B1-selective inhibitor TMS. CYP1B1 also promoted AEC proliferation and migration in both BEAS-2B and HBEC3-KT cell lines, akin to the effects reported for breast cancer cell lines overexpressing CYP1B1 (Kwon et al. 2016). The decrease in cytotoxicity and promotion of cell proliferation and migration supports the hypothesis that CYP1B1 induction in AECs may function to limit injury and inflammation and promote repair.

ERS is a conserved response to the accumulation of unfolded or misfolded proteins due to various causes (oxidative stress, ER calcium deficiency, viral infection, mutations, etc) (Burman et al. 2018; Nguyen et al. 2020). The unfolded protein response (UPR) is mediated by three major pathways: Pancreatic endoplasmic reticulum kinase (PERK/EIF2αK3), inositol-requiring enzyme 1 (IRE1α/β), and activating transcription factor 6 (ATF6) (Burman et al. 2018; Nguyen et al. 2020). Disrupted ERS/UPR signaling is implicated in various pathologies including pulmonary fibrosis, asthma, and lung cancer (Martino et al. 2013; Marciniak 2017; Burman et al. 2018). Our previous work demonstrated that the induction (during stress) and stable overexpression of TRPV3 by/in AECs conferred resistance to ERS manifesting as attenuated cell death caused by WSPM, whereas inhibiting TRPV3 exacerbated these effects (Nguyen et al. 2020). Here, ERS was exacerbated by inhibiting CYP1B1 in HBEC3-KT cells in response to WSPM treatment, while CYP1B1 overexpression was associated with a shift in the relative induction of pro-apoptotic (ie, *DDIT3*) vs. adaptive (*ATF3*, *TRPV3*, *HSPA1A*, *XBP1*-splicing) gene expression following WSPM treatment. Since the intent of the ERS response and UPR is ultimately to protect cells, but excessive or persistent ERS results in cell damage and/or death (Tsai and Weissman 2010; Zhang et al. 2023), the effects of CYP1B1 on ERS/UPR signaling may partially explain the beneficial impact of CYP1B1 on cytotoxicity and AEC proliferation/monolayer repair.

**Fig. 12.**
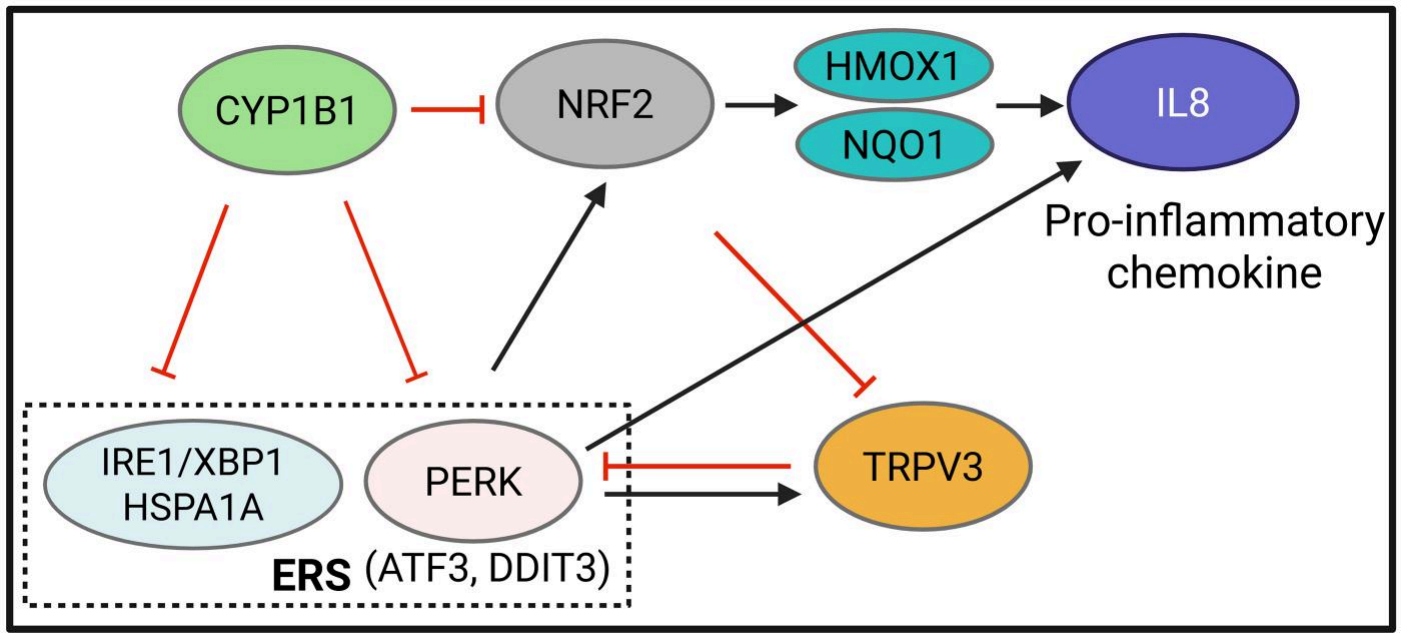
Scheme illustrating how CYP1B1 may protect AECs from WSPM-induced injury.

There is also a causal relationship between oxidative stress and ERS (Zhang et al. 2019, 2023) and studies have shown that ERS activates NRF2, particularly via PERK/EIF2αK3 (Cullinan and Diehl 2004). Further, increased expression of NRF2 inhibits ERS (Li et al. 2020; Naresh Amin et al. 2021). KEAP1/NRF2 is the primary signaling pathway involved in regulating oxidative stress and resistance. NRF2 is bound to KEAP1 in the cytoplasm under normal circumstances. Oxidative stress oxidizes cysteine residues and causes a conformational change in KEAP1 liberating NRF2 (and preventing its degradation) which then enters the nucleus to activate downstream target gene expression for proteins involved in glutathione synthesis and other antioxidant processes (Ma 2013). NRF2 has been shown to protect against cecal ligation and puncture (CLP)-induced and PM_2.5_-induced lung injury (Luo et al. 2022; Wang et al. 2022). Here, WSPM treatment increased NRF2 stability and target gene expression to a greater extent when CYP1B1 was inhibited in HBEC3-KT cells, versus a decrease in *HMOX1* and *NQO1* mRNA induction in B2B CYP1B1OE cells exposed to WSPM. These data suggest that NRF2-regulated antioxidant pathways are modulated by CYP1B1, supporting the hypothesis that regulating the NRF2-ERS nexus is a mechanism by which CYP1B1 protects AECs from WSPM-induced injury, as outlined by (Fig. 12). An effect of the NRF2-ERS nexus on CYP1B1 is also likely involved.

CYP1B1 also regulated *IL8* mRNA expression by AECs, suggesting a potential anti-inflammatory mechanism in the context of acute injury. Consistent with this idea, we found that the baseline levels of neutrophils and neutrophil chemoattracting *Cxcl1* and *Cxcl2* were increased in *Cyp1b1*-deficient mice. Unexpectedly, an exacerbation of neutrophilia after WSPM treatment did not occur in these mice but did in wild-type mice. This observation suggests that the pulmonary inflammatory response in *Cyp1b1*-deficient mice may be independent of *Cyp1b1* expression/function. One possible explanation is that absence of *Cyp1b1* induces compensatory changes in the lung. This may include altered chemokine regulation or desensitization of neutrophil recruitment pathways, thereby limiting neutrophil influx following WSPM exposure. Increased basal and induced *Cxcl1* and *Cxcl2* mRNA expression were also observed in *Cyp1b1^−/−^* MTECs compared with wild-type-derived MTECs. Consistent with a previous study, we also observed increased *CYP1A* gene mRNA expression, especially *CYP1A1*, in both *Cyp1b1^−/−^* mice and MTECs (Veith et al. 2018). Several reports have shown that pulmonary CYP1A enzymes (CYP1A1 and CYP1A2) may attenuate oxidative stress and lung injury induced by hyperoxia exposure (Lingappan et al. 2014, 2017; Wang et al. 2015; Veith et al. 2018; Stading et al. 2021). The compensatory upregulation of CYP1A, which has known protective roles against oxidative lung injury, may explain the absence of marked differences in inflammatory outcomes between wild-type and *Cyp1b1*-deficient mice following pine WSPM exposure.

An intriguing finding was that the beneficial aspects of CYP1B1 on ERS, NRF2, and proinflammatory cytokine gene expression primarily manifested in the context of injury. Other modifiers of CYP1B1 should be tested to better understand the role of CYP1B1 and to validate the current results. In addition, the mechanism by which CYP1B1 modulates ERS, NRF2 signaling, and inflammation is unresolved. CYP enzymes including CYP1B1, CYP1A1, CYP2C8 catalyze ω-6 and ω-3 fatty acids into biologically active eicosanoids including epoxyeicosatrienoic acids (EETs), hydroxyeicosatetraenoic acids (HETEs), epoxyoctadecenoic acids (EpOMEs), and epoxyeicosatetraenoic acids (EEQs) (Panigrahy et al. 2010; Sun et al. 2023; Zhang et al. 2023). Several of these lipid mediators, including EETs and EpOMEs, can activate or inhibit TRP ion channels including TRPA1 and TRPV1, which have been implicated in pulmonary injury and inflammation (Schafer et al. 2020; Sun et al. 2023). As illustrated for CYP2C8 (Almestica-Roberts et al. 2024), these lipids produced by CYP1B1 (and CYP1A1 or 1A2) may similarly modulate TRP channel activity to modulate WSPM-induced lung injury. However, studies are needed to confirm/refute this hypothetical paradigm.

## Conclusions

This study shows that CYP1B1 is associated with attenuation of WSPM-induced AEC injury that via modifications to pathological features of the ERS/UPR, NRF2 signaling, oxidative stress, and inflammatory responses. The results suggest that the induction of CYP1B1 by air pollutants such as WSPM or other forms of injury may constitute a normal protective response that promotes AEC proliferation and migration, possibly contributing to wound repair of a damaged airway epithelium. This study provides new insights into this protective role for CYP1B1 and potential mechanisms driving these effects in AECs. Additional experiments are needed to determine the scope of CYP1B1 protection against other stimuli and overlapping roles for CYPs such as 1A1 and 1A2, and whether the effects of CYP1B1 are related to specific metabolic processes, such as altered lipid mediator production/breakdown.

## Supplementary Material

kfag003_Supplementary_Data
