# Supplementary material for: CYP1B1 modulates stress and repair pathways in airway cells challenged by wood smoke particles
Source: Toxicol Sci. 2026 Jan 25;209(2):kfag003. doi: 10.1093/toxsci/kfag003 (PMC13397018; doi:10.1093/toxsci/kfag003)
Supplement: kfag003_Supplementary_Data [file kfag003_supplementary_data.docx]

**Supplementary Material**

**Title:** CYP1B1 Modulates Stress and Repair Pathways in Airway Cells Challenged by Wood Smoke Particles

**Authors:** Lili Sun, Marysol Almestica-Roberts, Nam D. Nguyen, Jacob Cowley, Erin Gail Romero, Samantha N. Serna, Peng Zhang, Changshan Niu, Tosifa A. Memon, Cassandra E. Deering-Rice, and Christopher A. Reilly

**Affiliations:** (LS, EGR, CEDR, CAR) Department of Pharmacology and Toxicology, Center for Human Toxicology, University of Utah, 30 S 2000 E, 307 L.S. Skaggs Hall, Salt Lake City, Utah 84112, United States

(MAR, NDN, JC, SNS, PZ, CN, TAM) Department of Pharmacology and Toxicology, University of Utah, 30 S 2000 E, Room 201 Skaggs Hall, Salt Lake City, Utah 84112, United States

**Corresponding author:**

Christopher A. Reilly, Ph.D.

Department of Pharmacology and Toxicology, Center for Human Toxicology, University of Utah

30 S 2000 E, Room 201 Skaggs Building

Salt Lake City, Utah 84112, United States

Phone: 801-581-5236

Email: [Chris.Reilly@pharm.utah.edu](mailto:Chris.Reilly@pharm.utah.edu)

**Table S1** Primer sequences for CYP1B1 sub-cloning.

| Oligonucleotides A | 5’-TTGGTACCCACCATGGGCACCAGCCTCAG-3’ |
| --- | --- |
| Oligonucleotides B | 5’-GTGGATCCTCAATGGTGATGGTGATGATGACC-3’ |

**Table S2** Primer sequences for *Cyp1b1* genotyping.

| Marker | Primer | Product size (bp) |
| --- | --- | --- |
| *Cyp1b1* WT | 5′-TTTGCCTGTCACCATTCCAC-3′ | 365 |
|  | 5′-ACGACTTGGGCTTAATGGTC-3′ |  |
| *Cyp1b1^-/-^* | 5′-TGAATGAACTGCAGGACGAG-3′ | 460 |
|  | 5′-CCACAGTCGATGAATCCAGA-3′ |  |
| Wild-type mice (*Cyp1b1^+/+^*) have a single band at 365 bp, knockout mice (*Cyp1b1^−/−^*) a single band at 460 bp, and the heterozygote (*Cyp1b1^+/−^*) both bands. | | |

**Table S3**. TaqMan probes for qPCR analysis of biomarker genes.

| **Human Genes** | **Catalog Number** |  | **Mouse Genes** | **Catalog Number** |
| --- | --- | --- | --- | --- |
| β2M | Hs00187842_m1 |  | *Gapdh* | Mm99999915_g1 |
| TRPV3 | Hs00376854_m1 |  | *Trpv3* | Mm0045003_m1 |
| TRPA1 | Hs00175798_m1 |  | *Trpa1* | Mm01227437_m1 |
| CYP1A1 | Hs00153120_m1 |  | *Cyp1a1* | Mm00487218_m1 |
| CYP1A2 | Hs00167927_m1 |  | *Cyp1a2* | Mm00487224_m1 |
| CYP1B1 | Hs00164383_m1 |  | *Cyp1b1* | Mm00487229_m1 |
| MUC5AC | Hs01365616_m1 |  | *Muc5ac* | Mm01276718_m1 |
| HMOX1 | Hs01110250_m1 |  | *Hmox1* | Mm00516005_m1 |
| DDIT3 | Hs00358796_g1 |  | *Ddit3* | Mm01135937_g1 |
| ATF3 | Hs00231069_m1 |  |  |  |
| HSPA1A | Hs00359163_s1 |  |  |  |
| NFE2L2 | Hs00975961_g1 |  |  |  |
| PTGR1 | Hs00400932_m1 |  |  |  |
| NQO1 | Hs00168547_m1 |  |  |  |
| GCLC | Hs00155249_m1 |  |  |  |
| KEAP1 | Hs00202227_m1 |  |  |  |

**Table S4**. Detailed *p*-value information for all figures.

| **Figure No.** | Comparisons | Significant Summary | *p*-value |
| --- | --- | --- | --- |
| Figure 1C | Vehicle vs. Pine (20 μg/cm^2^) |  |  |
|  | 0 h | ns | >0.9999 |
|  | 1 h | * | 0.0442 |
|  | 2 h | **** | <0.0001 |
|  | 4 h | **** | <0.0001 |
|  | 6 h | *** | 0.0009 |
|  | 8 h | ** | 0.0099 |
|  | 12 h | ns | >0.9999 |
|  | 16 h | ns | >0.9999 |
|  | 20 h | ns | 0.4153 |
|  | 24 h | **** | <0.0001 |
| Figure 2A | BEAS-2B FRT vs. B2B CYP1B1 OE |  |  |
|  | 0 μg/cm^2^ | ns | >0.9999 |
|  | 1 μg/cm^2^ | **** | <0.0001 |
|  | 2.5 μg/cm^2^ | **** | <0.0001 |
|  | 5 μg/cm^2^ | **** | <0.0001 |
|  | 10 μg/cm^2^ | * | 0.0119 |
|  | 20 μg/cm^2^ | * | 0.0142 |
|  | 30 μg/cm^2^ | **** | <0.0001 |
|  | 40 μg/cm^2^ | **** | <0.0001 |
|  | 50 μg/cm^2^ | ns | >0.9999 |
| Figure 2B | 3KT m pine vs. 3KT m pine +TMS (1 μM) |  |  |
|  | 0 μg/cm^2^ | ns | >0.9999 |
|  | 1 μg/cm^2^ | **** | <0.0001 |
|  | 2.5 μg/cm^2^ | **** | <0.0001 |
|  | 5 μg/cm^2^ | **** | <0.0001 |
|  | 10 μg/cm^2^ | ns | 0.0903 |
|  | 20 μg/cm^2^ | ns | >0.9999 |
|  | 30 μg/cm^2^ | ns | >0.9999 |
|  | 40 μg/cm^2^ | ns | >0.9999 |
|  | 50 μg/cm^2^ | ns | >0.9999 |
| Figure 5A | Vehicle:Control vs. Vehicle:Pine (20 μg/cm2) | **** | <0.0001 |
|  | Vehicle:Control vs. TMS (10μM):Control | ns | 0.9758 |
|  | Vehicle:Control vs. TMS (10μM):Pine (20 μg/cm2) | ns | 0.9987 |
|  | Vehicle:Pine (20 μg/cm2) vs. TMS (10μM):Control | **** | <0.0001 |
|  | Vehicle:Pine (20 μg/cm2) vs. TMS (10μM):Pine (20 μg/cm2) | **** | <0.0001 |
|  | TMS (10μM):Control vs. TMS (10μM):Pine (20 μg/cm2) | ns | 0.9935 |
| Figure 5B | Vehicle:Control vs. Vehicle:Pine (20 μg/cm2) | **** | <0.0001 |
|  | Vehicle:Control vs. TMS (10μM):Control | ns | >0.9999 |
|  | Vehicle:Control vs. TMS (10μM):Pine (20 μg/cm2) | **** | <0.0001 |
|  | Vehicle:Pine (20 μg/cm2) vs. TMS (10μM):Control | **** | <0.0001 |
|  | Vehicle:Pine (20 μg/cm2) vs. TMS (10μM):Pine (20 μg/cm2) | ns | 0.2717 |
|  | TMS (10μM):Control vs. TMS (10μM):Pine (20 μg/cm2) | **** | <0.0001 |
| Figure 5C | Vehicle:Control vs. Vehicle:Pine (20 μg/cm2) | **** | <0.0001 |
|  | Vehicle:Control vs. TMS (10μM):Control | ns | >0.9999 |
|  | Vehicle:Control vs. TMS (10μM):Pine (20 μg/cm2) | **** | <0.0001 |
|  | Vehicle:Pine (20 μg/cm2) vs. TMS (10μM):Control | **** | <0.0001 |
|  | Vehicle:Pine (20 μg/cm2) vs. TMS (10μM):Pine (20 μg/cm2) | **** | <0.0001 |
|  | TMS (10μM):Control vs. TMS (10μM):Pine (20 μg/cm2) | **** | <0.0001 |
| Figure 5D | Vehicle:Control vs. Vehicle:Pine (20 μg/cm2) | **** | <0.0001 |
|  | Vehicle:Control vs. TMS (10μM):Control | ns | >0.9999 |
|  | Vehicle:Control vs. TMS (10μM):Pine (20 μg/cm2) | **** | <0.0001 |
|  | Vehicle:Pine (20 μg/cm2) vs. TMS (10μM):Control | **** | <0.0001 |
|  | Vehicle:Pine (20 μg/cm2) vs. TMS (10μM):Pine (20 μg/cm2) | **** | <0.0001 |
|  | TMS (10μM):Control vs. TMS (10μM):Pine (20 μg/cm2) | **** | <0.0001 |
| Figure 5E | Vehicle:Control vs. Vehicle:Pine (20 μg/cm2) | ns | >0.9999 |
|  | Vehicle:Control vs. TMS (10μM):Control | ns | >0.9999 |
|  | Vehicle:Control vs. TMS (10μM):Pine (20 μg/cm2) | **** | <0.0001 |
|  | Vehicle:Pine (20 μg/cm2) vs. TMS (10μM):Control | ns | >0.9999 |
|  | Vehicle:Pine (20 μg/cm2) vs. TMS (10μM):Pine (20 μg/cm2) | **** | <0.0001 |
|  | TMS (10μM):Control vs. TMS (10μM):Pine (20 μg/cm2) | **** | <0.0001 |
| Figure 5F | Vehicle:Control vs. Vehicle:Pine (20 μg/cm2) | **** | <0.0001 |
|  | Vehicle:Control vs. TMS (10μM):Control | ns | 0.9908 |
|  | Vehicle:Control vs. TMS (10μM):Pine (20 μg/cm2) | **** | <0.0001 |
|  | Vehicle:Pine (20 μg/cm2) vs. TMS (10μM):Control | **** | <0.0001 |
|  | Vehicle:Pine (20 μg/cm2) vs. TMS (10μM):Pine (20 μg/cm2) | **** | <0.0001 |
|  | TMS (10μM):Control vs. TMS (10μM):Pine (20 μg/cm2) | **** | <0.0001 |
| Figure 6A | Vehicle:Control vs. Vehicle:Pine (20 μg/cm2) | ns | 0.4787 |
|  | Vehicle:Control vs. TMS (10μM):Control | ns | 0.3139 |
|  | Vehicle:Control vs. TMS (10μM):Pine (20 μg/cm2) | ns | 0.9623 |
|  | Vehicle:Pine (20 μg/cm2) vs. TMS (10μM):Control | ns | 0.9827 |
|  | Vehicle:Pine (20 μg/cm2) vs. TMS (10μM):Pine (20 μg/cm2) | ns | 0.7396 |
|  | TMS (10μM):Control vs. TMS (10μM):Pine (20 μg/cm2) | ns | 0.5395 |
| Figure 6B | Vehicle:Control vs. Vehicle:Pine (20 μg/cm2) | **** | <0.0001 |
|  | Vehicle:Control vs. TMS (10μM):Control | ns | 0.9413 |
|  | Vehicle:Control vs. TMS (10μM):Pine (20 μg/cm2) | **** | <0.0001 |
|  | Vehicle:Pine (20 μg/cm2) vs. TMS (10μM):Control | **** | <0.0001 |
|  | Vehicle:Pine (20 μg/cm2) vs. TMS (10μM):Pine (20 μg/cm2) | ns | 0.8504 |
|  | TMS (10μM):Control vs. TMS (10μM):Pine (20 μg/cm2) | **** | <0.0001 |
| Figure 6C | Vehicle:Control vs. Vehicle:Pine (20 μg/cm2) | **** | <0.0001 |
|  | Vehicle:Control vs. TMS (10μM):Control | ns | 0.6212 |
|  | Vehicle:Control vs. TMS (10μM):Pine (20 μg/cm2) | **** | <0.0001 |
|  | Vehicle:Pine (20 μg/cm2) vs. TMS (10μM):Control | **** | <0.0001 |
|  | Vehicle:Pine (20 μg/cm2) vs. TMS (10μM):Pine (20 μg/cm2) | *** | 0.0004 |
|  | TMS (10μM):Control vs. TMS (10μM):Pine (20 μg/cm2) | **** | <0.0001 |
| Figure 6D | Vehicle:Control vs. Vehicle:Pine (20 μg/cm2) | **** | <0.0001 |
|  | Vehicle:Control vs. TMS (10μM):Control | ns | >0.9999 |
|  | Vehicle:Control vs. TMS (10μM):Pine (20 μg/cm2) | **** | <0.0001 |
|  | Vehicle:Pine (20 μg/cm2) vs. TMS (10μM):Control | **** | <0.0001 |
|  | Vehicle:Pine (20 μg/cm2) vs. TMS (10μM):Pine (20 μg/cm2) | *** | 0.0002 |
|  | TMS (10μM):Control vs. TMS (10μM):Pine (20 μg/cm2) | **** | <0.0001 |
| Figure 6E | Vehicle:Control vs. Vehicle:Pine (20 μg/cm2) | *** | 0.0002 |
|  | Vehicle:Control vs. TMS (10μM):Control | ns | 0.6912 |
|  | Vehicle:Control vs. TMS (10μM):Pine (20 μg/cm2) | **** | <0.0001 |
|  | Vehicle:Pine (20 μg/cm2) vs. TMS (10μM):Control | **** | <0.0001 |
|  | Vehicle:Pine (20 μg/cm2) vs. TMS (10μM):Pine (20 μg/cm2) | ns | 0.0689 |
|  | TMS (10μM):Control vs. TMS (10μM):Pine (20 μg/cm2) | **** | <0.0001 |
| Figure 6F | Vehicle:Control vs. Vehicle:Pine (20 μg/cm2) | **** | <0.0001 |
|  | Vehicle:Control vs. TMS (10μM):Control | ns | 0.9784 |
|  | Vehicle:Control vs. TMS (10μM):Pine (20 μg/cm2) | **** | <0.0001 |
|  | Vehicle:Pine (20 μg/cm2) vs. TMS (10μM):Control | **** | <0.0001 |
|  | Vehicle:Pine (20 μg/cm2) vs. TMS (10μM):Pine (20 μg/cm2) | ns | 0.9959 |
|  | TMS (10μM):Control vs. TMS (10μM):Pine (20 μg/cm2) | **** | <0.0001 |
| Figure 7A | B2B FRT:Control vs. B2B FRT:Pine (10 μg/cm2) | ** | 0.0024 |
|  | B2B FRT:Control vs. B2B CYP1B1 OE:Control | ns | 0.9801 |
|  | B2B FRT:Control vs. B2B CYP1B1 OE:Pine (10 μg/cm2) | *** | 0.0008 |
|  | B2B FRT:Pine (10 μg/cm2) vs. B2B CYP1B1 OE:Control | ** | 0.0016 |
|  | B2B FRT:Pine (10 μg/cm2) vs. B2B CYP1B1 OE:Pine (10 μg/cm2) | ns | 0.7208 |
|  | B2B CYP1B1 OE:Control vs. B2B CYP1B1 OE:Pine (10 μg/cm2) | *** | 0.0005 |
| Figure 7B | B2B FRT:Control vs. B2B FRT:Pine (10 μg/cm2) | **** | <0.0001 |
|  | B2B FRT:Control vs. B2B CYP1B1 OE:Control | ns | 0.8454 |
|  | B2B FRT:Control vs. B2B CYP1B1 OE:Pine (10 μg/cm2) | **** | <0.0001 |
|  | B2B FRT:Pine (10 μg/cm2) vs. B2B CYP1B1 OE:Control | **** | <0.0001 |
|  | B2B FRT:Pine (10 μg/cm2) vs. B2B CYP1B1 OE:Pine (10 μg/cm2) | * | 0.0222 |
|  | B2B CYP1B1 OE:Control vs. B2B CYP1B1 OE:Pine (10 μg/cm2) | **** | <0.0001 |
| Figure 7C | B2B FRT:Control vs. B2B FRT:Pine (10 μg/cm2) | **** | <0.0001 |
|  | B2B FRT:Control vs. B2B CYP1B1 OE:Control | * | 0.0207 |
|  | B2B FRT:Control vs. B2B CYP1B1 OE:Pine (10 μg/cm2) | **** | <0.0001 |
|  | B2B FRT:Pine (10 μg/cm2) vs. B2B CYP1B1 OE:Control | **** | <0.0001 |
|  | B2B FRT:Pine (10 μg/cm2) vs. B2B CYP1B1 OE:Pine (10 μg/cm2) | * | 0.0473 |
|  | B2B CYP1B1 OE:Control vs. B2B CYP1B1 OE:Pine (10 μg/cm2) | **** | <0.0001 |
| Figure 7D | B2B FRT:Control vs. B2B FRT:Pine (10 μg/cm2) | **** | <0.0001 |
|  | B2B FRT:Control vs. B2B CYP1B1 OE:Control | ns | 0.9998 |
|  | B2B FRT:Control vs. B2B CYP1B1 OE:Pine (10 μg/cm2) | *** | 0.0002 |
|  | B2B FRT:Pine (10 μg/cm2) vs. B2B CYP1B1 OE:Control | **** | <0.0001 |
|  | B2B FRT:Pine (10 μg/cm2) vs. B2B CYP1B1 OE:Pine (10 μg/cm2) | * | 0.0114 |
|  | B2B CYP1B1 OE:Control vs. B2B CYP1B1 OE:Pine (10 μg/cm2) | *** | 0.0002 |
| Figure 7E | B2B FRT:Control vs. B2B FRT:Pine (10 μg/cm2) | **** | <0.0001 |
|  | B2B FRT:Control vs. B2B CYP1B1 OE:Control | ns | 0.5602 |
|  | B2B FRT:Control vs. B2B CYP1B1 OE:Pine (10 μg/cm2) | **** | <0.0001 |
|  | B2B FRT:Pine (10 μg/cm2) vs. B2B CYP1B1 OE:Control | **** | <0.0001 |
|  | B2B FRT:Pine (10 μg/cm2) vs. B2B CYP1B1 OE:Pine (10 μg/cm2) | ns | 0.9191 |
|  | B2B CYP1B1 OE:Control vs. B2B CYP1B1 OE:Pine (10 μg/cm2) | **** | <0.0001 |
| Figure 7F | B2B FRT:Control vs. B2B FRT:Pine (10 μg/cm2) | **** | <0.0001 |
|  | B2B FRT:Control vs. B2B CYP1B1 OE:Control | ns | 0.0507 |
|  | B2B FRT:Control vs. B2B CYP1B1 OE:Pine (10 μg/cm2) | **** | <0.0001 |
|  | B2B FRT:Pine (10 μg/cm2) vs. B2B CYP1B1 OE:Control | **** | <0.0001 |
|  | B2B FRT:Pine (10 μg/cm2) vs. B2B CYP1B1 OE:Pine (10 μg/cm2) | ns | 0.0694 |
|  | B2B CYP1B1 OE:Control vs. B2B CYP1B1 OE:Pine (10 μg/cm2) | **** | <0.0001 |
| Figure 8B | Vehicle:Control vs. Vehicle:Pine (20 μg/cm2) | **** | <0.0001 |
|  | Vehicle:Control vs. TMS (10 μM):Control | ns | >0.9999 |
|  | Vehicle:Control vs. TMS (10 μM):Pine (20 μg/cm2) | **** | <0.0001 |
|  | Vehicle:Pine (20 μg/cm2) vs. TMS (10 μM):Control | **** | <0.0001 |
|  | Vehicle:Pine (20 μg/cm2) vs. TMS (10 μM):Pine (20 μg/cm2) | **** | <0.0001 |
|  | TMS (10 μM):Control vs. TMS (10 μM):Pine (20 μg/cm2) | **** | <0.0001 |
| Figure 10B | WT Saline vs. WT Pine (A-B) | * | 0.0338 |
|  | WT Saline vs. *Cyp1b1^-/-^* Saline (A-C) | ** | 0.0023 |
|  | WT Saline vs. *Cyp1b1^-/-^* Pine (A-D) | * | 0.0125 |
|  | WT Pine vs. *Cyp1b1^-/-^* Saline (B-C) | ns | 0.1907 |
|  | WT Pine vs. *Cyp1b1^-/-^* Pine (B-D) | ns | 0.5665 |
|  | *Cyp1b1^-/-^* Saline vs. *Cyp1b1^-/-^* Pine (C-D) | ns | 0.4672 |
| Figure 10C | WT Saline vs. WT Pine (A-B) | *** | 0.0002 |
|  | WT Saline vs. *Cyp1b1^-/-^* Saline (A-C) | * | 0.0309 |
|  | WT Saline vs. *Cyp1b1^-/-^* Pine (A-D) | ns | 0.1416 |
|  | WT Pine vs. *Cyp1b1^-/-^* Saline (B-C) | ns | 0.9883 |
|  | WT Pine vs. *Cyp1b1^-/-^* Pine (B-D) | ns | 0.386 |
|  | *Cyp1b1^-/-^* Saline vs. *Cyp1b1^-/-^* Pine (C-D) | ns | 0.5589 |
| Figure 10D | WT Saline vs. WT Pine (A-B) | ns | 0.9866 |
|  | WT Saline vs. *Cyp1b1^-/-^* Saline (A-C) | ns | 0.1139 |
|  | WT Saline vs. *Cyp1b1^-/-^* Pine (A-D) | ns | 0.154 |
|  | WT Pine vs. *Cyp1b1^-/-^* Saline (B-C) | ns | 0.0599 |
|  | WT Pine vs. *Cyp1b1^-/-^* Pine (B-D) | ns | 0.0856 |
|  | *Cyp1b1^-/-^* Saline vs. *Cyp1b1^-/-^* Pine (C-D) | ns | >0.9999 |
| Figure 10E | WT Saline vs. WT Pine (A-B) | ns | 0.4838 |
|  | WT Saline vs. *Cyp1b1^-/-^* Saline (A-C) | ns | 0.6845 |
|  | WT Saline vs. *Cyp1b1^-/-^* Pine (A-D) | ns | 0.7464 |
|  | WT Pine vs. *Cyp1b1^-/-^* Saline (B-C) | ns | 0.9864 |
|  | WT Pine vs. *Cyp1b1^-/-^* Pine (B-D) | ns | 0.9813 |
|  | *Cyp1b1^-/-^* Saline vs. *Cyp1b1^-/-^* Pine (C-D) | ns | >0.9999 |
| Figure 11A | WT:Control vs. WT:Pine (5 μg/cm2) | ns | 0.7485 |
|  | WT:Control vs. *Cyp1b1^-/-^*:Control | *** | 0.0001 |
|  | WT:Control vs. *Cyp1b1^-/-^*:Pine (5 μg/cm2) | **** | <0.0001 |
|  | WT:Pine (5 μg/cm2) vs. *Cyp1b1^-/-^*:Control | *** | 0.0003 |
|  | WT:Pine (5 μg/cm2) vs. *Cyp1b1^-/-^*:Pine (5 μg/cm2) | **** | <0.0001 |
|  | *Cyp1b1^-/-^*:Control vs. *Cyp1b1^-/-^*:Pine (5 μg/cm2) | * | 0.03 |
| Figure 11B | WT:Control vs. WT:Pine (5 μg/cm2) | ** | 0.0014 |
|  | WT:Control vs. *Cyp1b1^-/-^*:Control | ns | 0.9145 |
|  | WT:Control vs. *Cyp1b1^-/-^*:Pine (5 μg/cm2) | **** | <0.0001 |
|  | WT:Pine (5 μg/cm2) vs. *Cyp1b1^-/-^*:Control | ** | 0.0029 |
|  | WT:Pine (5 μg/cm2) vs. *Cyp1b1^-/-^*:Pine (5 μg/cm2) | *** | 0.0009 |
|  | *Cyp1b1^-/-^*:Control vs. *Cyp1b1^-/-^*:Pine (5 μg/cm2) | **** | <0.0001 |
| Figure 11C | WT:Control vs. WT:Pine (5 μg/cm2) | *** | 0.0004 |
|  | WT:Control vs. *Cyp1b1^-/-^*:Control | ns | 0.9495 |
|  | WT:Control vs. *Cyp1b1^-/-^*:Pine (5 μg/cm2) | * | 0.0242 |
|  | WT:Pine (5 μg/cm2) vs. *Cyp1b1^-/-^*:Control | *** | 0.0002 |
|  | WT:Pine (5 μg/cm2) vs. *Cyp1b1^-/-^*:Pine (5 μg/cm2) | * | 0.0315 |
|  | *Cyp1b1^-/-^*:Control vs. *Cyp1b1^-/-^*:Pine (5 μg/cm2) | * | 0.0119 |
| Figure 11D | WT:Control vs. WT:Pine (5 μg/cm2) | ** | 0.0011 |
|  | WT:Control vs. *Cyp1b1^-/-^*:Control | ns | 0.9836 |
|  | WT:Control vs. *Cyp1b1^-/-^*:Pine (5 μg/cm2) | * | 0.0195 |
|  | WT:Pine (5 μg/cm2) vs. *Cyp1b1^-/-^*:Control | *** | 0.0008 |
|  | WT:Pine (5 μg/cm2) vs. *Cyp1b1^-/-^*:Pine (5 μg/cm2) | ns | 0.1583 |
|  | *Cyp1b1^-/-^*:Control vs. *Cyp1b1^-/-^*:Pine (5 μg/cm2) | * | 0.0121 |
| Figure 11E | WT:Control vs. WT:Pine (5 μg/cm2) | ns | 0.4981 |
|  | WT:Control vs. *Cyp1b1^-/-^*:Control | ns | >0.9999 |
|  | WT:Control vs. *Cyp1b1^-/-^*:Pine (5 μg/cm2) | *** | 0.0007 |
|  | WT:Pine (5 μg/cm2) vs. *Cyp1b1^-/-^*:Control | ns | 0.5217 |
|  | WT:Pine (5 μg/cm2) vs. *Cyp1b1^-/-^*:Pine (5 μg/cm2) | ** | 0.0034 |
|  | *Cyp1b1^-/-^*:Control vs. *Cyp1b1^-/-^*:Pine (5 μg/cm2) | *** | 0.0007 |
| Figure 11F | WT:Control vs. WT:Pine (5 μg/cm2) | ns | 0.9148 |
|  | WT:Control vs. *Cyp1b1^-/-^*:Control | ns | 0.996 |
|  | WT:Control vs. *Cyp1b1^-/-^*:Pine (5 μg/cm2) | ** | 0.0014 |
|  | WT:Pine (5 μg/cm2) vs. *Cyp1b1^-/-^*:Control | ns | 0.9622 |
|  | WT:Pine (5 μg/cm2) vs. *Cyp1b1^-/-^*:Pine (5 μg/cm2) | ** | 0.0014 |
|  | *Cyp1b1^-/-^*:Control vs. *Cyp1b1^-/-^*:Pine (5 μg/cm2) | *** | 0.0009 |

**Figure S1:** *TRPV3* mRNA was upregulated in both (**A**) BEAS-2B and (**B**) HBEC3-KT cell lines treated with pine WSPM (10 µg/cm^2^) for 24 hours. Data were normalized to the vehicle control for each cell type and are shown as the mean ± S.D. (n=3). Statistical testing was performed using two-tailed Student’s T-test. ^🞸^*P* < 0.05, ^🞸🞸🞸^*P* < 0.001. (**C**) Temporal changes in *TRPV3* mRNA expression in HBEC3-KT cells after pine WSPM treatment (20 µg/cm^2^). Each time point was normalized to 0-hour vehicle-treated HBEC3-KT cells and is presented as the mean ± S.D. (n=3). Statistical testing was performed using two-way ANOVA with Bonferroni’s multiple comparisons test. ^🞸^*p* < 0.05, ^🞸🞸🞸🞸^*p* < 0.0001.

**Figure S2: (A)** Expression of *CYP1B1* mRNA in human immortalized, cancer-derived, and primary airway epithelial cells representing proximal and distal airways. **(B**) Western blot and quantification of CYP1B1 protein in BEAS-2B FRT and CYP1B1 over-expressing cells. Data were normalized to the Flp-In BEAS-2B cell line and are presented as mean ± S.D. (n=3). Statistical testing was performed using two-tailed *Student’s T-test*. ^🞸🞸🞸^*p*< 0.001.

**Figure S3: (A)** CYP1B1 overexpression altered **(B)** *TRPV3* expression and exacerbated ERS in BEAS-2B cells in response to WSPM treatment, as evidenced by the upregulation of the biomarkers **(D)** *DDIT3*, **(E)** *HSPA1A* and **(F)** *XBP1* splicing but not (**C**) *ATF3*. Data were normalized to control BEAS-2B FRT cells and are presented as the mean ± S.D. (n=3). Statistical testing was performed using two-way ANOVA with Tukey’s multiple comparisons test. ^🞸^*p* < 0.05, ^🞸🞸^*p* < 0.001, ^🞸🞸🞸🞸^*p* < 0.0001.

**Figure S4:** Cytochrome P450 mRNA expression in the lung of wild-type C57Bl/6J and *Cyp1b1*-deficient mice treated with saline or pine WSPM via the oropharyngeal route: **(A)** *Cyp1a1*, **(B)** *Cyp1a2*, **(C)** *Cyp2j2*, and **(D)** *Cyp2f2.* Data were normalized to wild-type mice treated with saline and are shown as the mean ± S.E.M. (n=6). Statistical testing was performed using one-way ANOVA with Tukey’s multiple comparisons test. ^🞸^*p* < 0.05, ^🞸🞸^*p* < 0.01, ^🞸🞸🞸^*p* < 0.001.

**
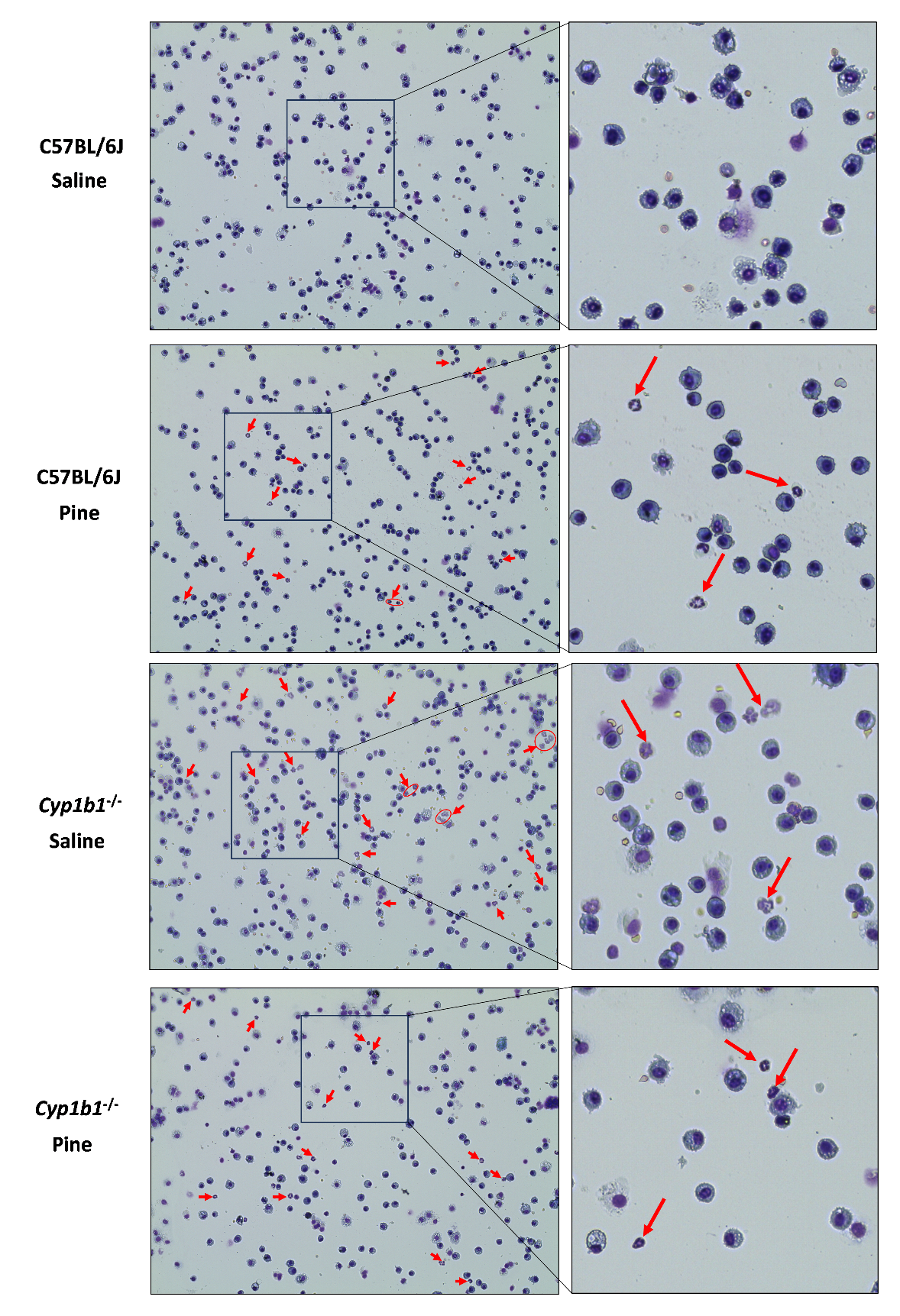
**

**Figure S5:** Representative images (20X) of cells recovered in BALF of wild-type C57Bl/6J and *Cyp1b1*-deficient mice treated with saline or pine WSPM. The callouts show expanded images of representative cells. Red arrows correspond to neutrophils.
